# Band 3–mediated *Plasmodium vivax* invasion is associated with transcriptional variation in *PvTRAg* genes

**DOI:** 10.3389/fcimb.2022.1011692

**Published:** 2022-09-30

**Authors:** Katlijn De Meulenaere, Surendra Kumar Prajapati, Elizabeth Villasis, Bart Cuypers, Johanna Helena Kattenberg, Bernadine Kasian, Moses Laman, Leanne J. Robinson, Dionicia Gamboa, Kris Laukens, Anna Rosanas-Urgell

**Affiliations:** ^1^ Department of Biomedical Sciences, Institute of Tropical Medicine Antwerp, Antwerp, Belgium; ^2^ Department of Computer Science, University of Antwerp, Antwerp, Belgium; ^3^ Department of Microbiology and Immunology, Uniformed Services University of the Health Sciences, Bethesda, MD, United States; ^4^ Laboratorio de Malaria, Laboratorios de Investigación y Desarrollo, Facultad de Ciencias y Filosofía, Universidad Peruana Cayetano Heredia, Lima, Peru; ^5^ Instituto de Medicina Tropical Alexander von Humboldt, Universidad Peruana Cayetano Heredia, Lima, Peru; ^6^ Vector-borne Diseases Unit, Papua New Guinea Institute for Medical Research, Madang, Papua New Guinea; ^7^ Population Health and Immunity Division, Walter and Eliza Hall Institute of Medical Research, Melbourne, VIC, Australia; ^8^ Department of Medical Biology, University of Melbourne, Melbourne, VIC, Australia; ^9^ Health Security and Disease Elimination, Burnet Institute, Melbourne, VIC, Australia; ^10^ Departamento de Ciencias Celulares y Moleculares, Facultad de Ciencias y Filosofía, Universidad Peruana Cayetano Heredia, Lima, Peru

**Keywords:** *P. vivax* (*Plasmodium vivax*), Southeast Asian Ovalocytosis (SAO), transcriptome, differential expression analysis, malaria, band 3, *ex vivo* invasion, RNA-seq

## Abstract

The *Plasmodium vivax* reticulocyte invasion process is still poorly understood, with only a few receptor-ligand interactions identified to date. Individuals with the Southeast Asian ovalocytosis (SAO) phenotype have a deletion in the band 3 protein on the surface of erythrocytes, and are reported to have a lower incidence of clinical *P. vivax* malaria. Based on this observation, band 3 has been put forward as a receptor for *P. vivax* invasion, although direct proof is still lacking. In this study, we combined functional *ex vivo* invasion assays and transcriptome sequencing to uncover a band 3–mediated invasion pathway in *P. vivax* and potential band 3 ligands. Invasion by *P. vivax* field isolates was 67%-71% lower in SAO reticulocytes compared with non-SAO reticulocytes. Reticulocyte invasion was decreased by 40% and 27%-31% when blocking with an anti-band 3 polyclonal antibody and a PvTRAg38 peptide, respectively. To identify new band 3 receptor candidates, we mRNA-sequenced schizont-stage isolates used in the invasion assays, and observed high transcriptional variability in multigene and invasion-related families. Transcriptomes of isolates with low or high dependency on band 3 for invasion were compared by differential expression analysis, which produced a list of band 3 ligand candidates with high representation of *PvTRAg* genes. Our *ex vivo* invasion assays have demonstrated that band 3 is a *P. vivax* invasion receptor and confirm previous *in vitro* studies showing binding between PvTRAg38 and band 3, although the lower and variable inhibition levels observed suggest the involvement of other ligands. By coupling transcriptomes and invasion phenotypes from the same isolates, we identified a list of band 3 ligand candidates, of which the overrepresented *PvTRAg* genes are the most promising for future research.

## Introduction

*Plasmodium vivax*, the most widespread human malaria species, is responsible for approximately one-third of all malaria cases outside Africa (WHO, 2021). It is more difficult to eliminate than *P. falciparum*, in part because of its unique biologic properties, which include hypnozoites (dormant forms), gametocyte formation during the first blood-stage cycle, and reticulocyte tropism, which results in lower peripheral parasite densities and cryptic cycles in the spleen and bone marrow (Silva-Filho et al., 2020; Kho et al., 2021). Although *P. vivax* is increasingly recognized as a cause of morbidity and mortality (Price et al., 2009; Battle and Baird, 2021) and represents a substantial economic burden (Devine et al., 2021), major knowledge gaps remain, including the molecular mechanism underlying reticulocyte invasion. A better understanding of receptor-ligand interactions involved in this process could provide new targets for vaccine development (Beeson et al., 2016; Tham et al., 2017).

Because *P. vivax* was thought to be absent in areas of Central Africa where the Duffy-negative blood type is predominant (Miller et al., 1976; Welch et al., 1977), it was long believed that *P. vivax* invasion was strictly mediated by the interaction between the human Duffy antigen receptor for chemokines (DARC, Duffy) and the *P. vivax* Duffy binding protein (PvDBP). This belief was supported by high inhibition of invasion in assays targeting DARC and PvDBP (Barnwell et al., 1989; Grimberg et al., 2007; Kanjee et al., 2020). Multiple studies, however, have since reported *P. vivax* infections in Duffy-negative individuals (Gunalan et al., 2018), indicating the existence of multiple host-parasite interactions and perhaps alternative pathways of entry into reticulocytes. Although the lack of a reliable continuous culture system for *P. vivax* remains a major challenge, new receptor-ligand interactions that occur during invasion have recently been described. Interactions between the parasite ligands *P. vivax* reticulocyte binding proteins 2b (PvRBP2b) and PvRBP2a and the human transferrin receptor 1 (TfR1, CD71) and CD98, respectively (Gruszczyk et al., 2018; Malleret et al., 2021), define the tropism of *P. vivax* for reticulocytes, as both receptors are highly abundant in young reticulocytes. Ligand candidates PvRBP1a, 1b, 2c and *P. vivax* erythrocyte binding protein (PvEBP) appear to contribute to this tropism *in vitro*, although their potential reticulocyte receptors are unknown (Han et al., 2016; Ntumngia et al., 2016; Gupta et al., 2017; Ntumngia et al., 2018). Basigin and Complement Receptor 1 (CR1) also function as receptors during *P. vivax* invasion, although their ligands on the parasite remain unidentified (Knuepfer et al., 2019; Prajapati et al., 2019).

In order to find new receptors involved in invasion, naturally occurring erythrocyte mutations that protect against *P. vivax* can help identify candidate receptors. Many red blood cell (RBC) polymorphisms associated with *P. falciparum* susceptibility are found at relatively high frequencies in Papua New Guinea, which has a high prevalence of malaria. Examples are alpha thalassemia, the CR1 exon 22 polymorphism, and the glycophorin C (GYPC) exon 3 deletion (Gerbich negative blood type) (Williams, 2006). The effect of these mutations on *P. vivax* susceptibility, however, has not been demonstrated (Williams et al., 1996; Patel et al., 2001; Rosanas-Urgell et al., 2012b; Para et al., 2018), or, as with CR1, has only been demonstrated at the population level  (Prajapati et al., 2019). On the other hand, Southeast Asian ovalocytosis (SAO), which is caused by a 27 base pair deletion in the band 3 protein on the surface of erythrocytes (SLC4A1Δ27), has been associated with a reduction of up to 52% in clinical *P. vivax* infections in Papua New Guinea (Rosanas-Urgell et al., 2012a). The SAO phenotype is most prevalent in coastal populations in Papua New Guinea (Mgone et al., 1996), but is also found in several Southeast Asian populations (Kimura et al., 1998; Wilder et al., 2009; Laosombat et al., 2010), where it closely correlates with malaria prevalence. This suggests a mechanism of protection that is achieved during the *P. vivax* invasion process, and that band 3 might be a receptor for invasion.

Band 3 is a transmembrane protein with 12 membrane-spanning regions and four extracellular loops. It is part of two membrane protein macrocomplexes: the band 3 complex and 4.1R complex (Arakawa et al., 2015; Lux, 2016); these include other (potential) membrane receptors, such as DARC, GYPC and Glycophorin A (GYPA). Band 3 functions as an anion transporter through the RBC membrane and contributes to the maintenance of RBC integrity by anchoring the cell membrane to the underlying cytoskeleton (Salomao et al., 2008). Because the SLC4A1Δ27 deletion in SAO RBCs is located at the start of the first transmembrane segment, the bond with ankyrin and thus the cytoskeleton is tighter, with erythrocytes adopting an elliptical shape and showing markedly increased rigidity. The SLC4A1Δ27 deletion is lethal in the homozygous state, and SAO is only found as a heterozygous genotype (Liu et al., 1994). SAO erythrocytes therefore contain about 50% mutant band 3 proteins, which aggregate predominantly to form hetero-tetramers and higher-order hetero-oligomers, inducing conformational changes in wildtype band 3. In normal RBCs, band 3 is mainly found in dimers and, to a lesser extent, tetramers (Sarabia et al., 1993; Kuma et al., 2002).

In *P. falciparum*, three ligands (PfMSP1, PfMSP9, and PfGAMA) bind to the band 3 receptor (Goel et al., 2003; Li et al., 2004). In *P. vivax*, although multiple ligand candidates have been identified in *in vitro* studies using recombinant proteins and mature RBCs (Zeeshan et al., 2014; Lu et al., 2022), it remains to be demonstrated whether these interactions are also involved in *P. vivax* invasion. A recent study using mass spectrometry identified binding between the GPI-anchored micronemal antigen (PvGAMA) and extracellular loop 5 of the band 3 protein (Lu et al., 2022). Antibodies specific for this loop also block the binding of recombinant PvGAMA to mature RBCs (Lu et al., 2022). In addition, *P. vivax* tryptophan-rich antigens (PvTRAgs) have been shown to interact with receptors (including band 3) on the surface of mature RBCs, suggesting a potential role in invasion (Zeeshan et al., 2014). The *PvTRAg* family has 40 genes (Auburn et al., 2016), which are expressed at the early ring stage or the late schizont stage of the erythrocytic cycle (Zhu et al., 2016). PvTRAg proteins are conserved proteins that induce cellular and humoral immune responses in *P. vivax*–infected patients, and ten members of this family have shown *in vitro* erythrocyte binding capacity to 5 different receptors (Zeeshan et al., 2013; Zeeshan et al., 2014; Wang et al., 2015). Each of these 10 PvTRAgs recognized more than 1 receptor and each receptor bound to more than 1 PvTRAg. Of the 10 proteins analyzed, PvTRAg38, PvTRAg36, and PvTRAg22 bound to band 3. PvTRAg38 and PvTRAg36 additionally bound to basigin, while PvTRAg22 also bound to another, unknown, receptor on the erythrocyte surface (Tyagi and Sharma, 2012; Zeeshan et al., 2014; Alam et al., 2016b; Rathore et al., 2017). Our understanding to date of PvTRAg binding capacities is based on *in vitro* experiments with PvTRAg recombinant proteins and receptor peptides and antibodies. Although these assays provide a strong indication of true binding between PvTRAgs and erythrocyte receptors and ligand redundancy, limited conclusions can be drawn for a number of reasons, including i) design issues related to the production of recombinant PvTRAg proteins and the risk of protein misfolding, ii) the use of receptor peptides which are not in their native conformation, and iii) the use of mature erythrocytes rather than reticulocytes. Based on current knowledge of band 3 binding in *P. vivax* and *P. falciparum*, we hypothesize that multiple *P. vivax* ligands (PvGAMA, PvTRAgs, and/or unknown ligands) are able to bind to band 3, possibly resulting in cooperative binding, alternative or redundant invasion pathways, or involvement in different steps of the invasion process.

**Figure 1:**
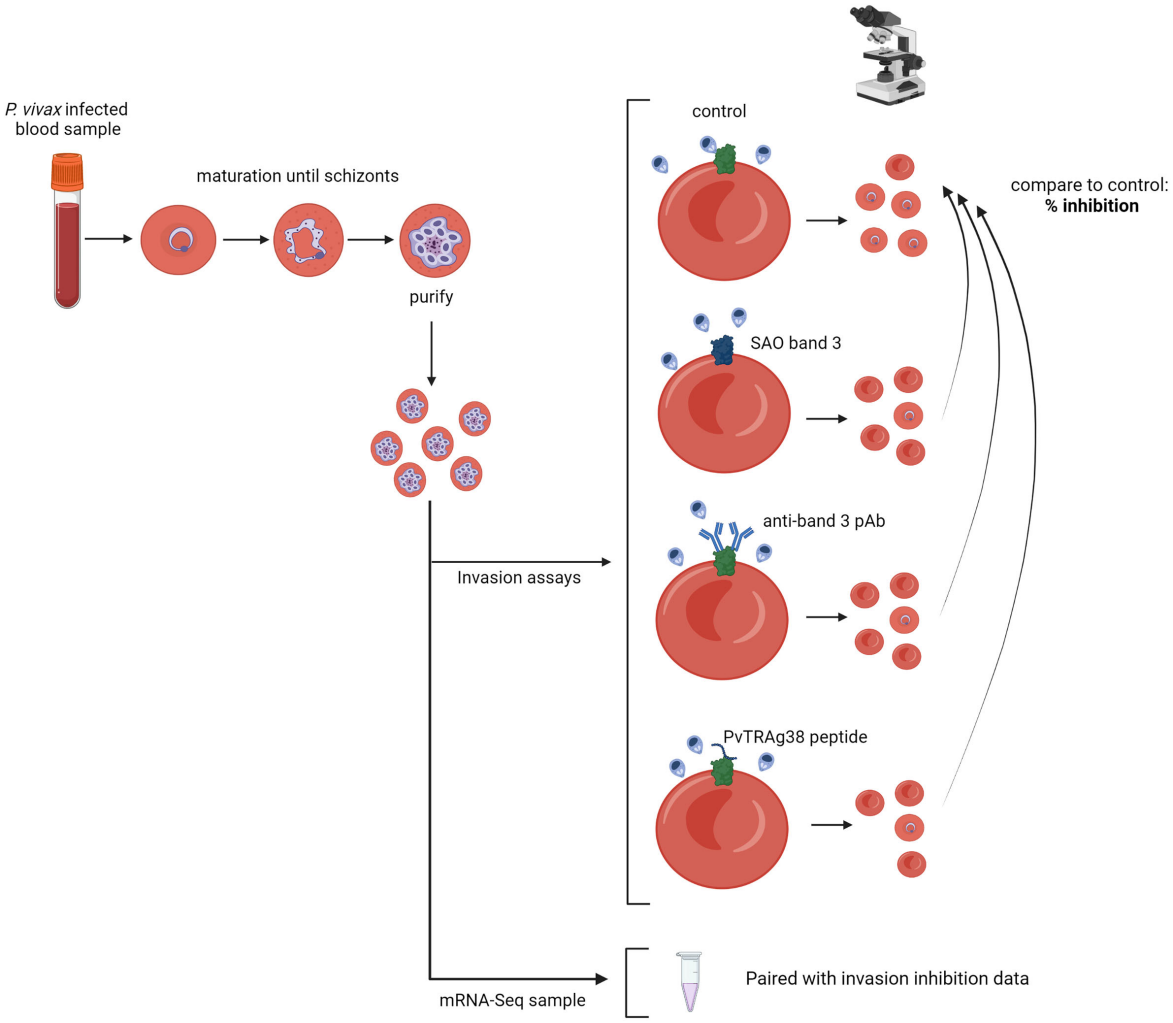
Schematic representation of the experimental approach. *P. vivax–*infected red blood cells are cultured until the majority have matured into schizonts, after which schizont stages are enriched and used for invasion assays; if enough schizonts are available, they are stored for subsequent mRNA sequencing. Three different invasion assay setups are used: invasion into SAO reticulocyte-enriched RBCs (reRBCs), band 3 blocking with a polyclonal antibody (pAb), and band 3 blocking with a PvTRAg38 peptide. The number of newly invaded ring- and young trophozoite-stage parasites is counted by light microscopy, and compared to the paired control (non-SAO reRBCs without antibody/peptide), resulting in a normalized percentage of invasion inhibition. Figure created with BioRender.com.

In this study, we aimed to confirm that *P. vivax* uses band 3 as a receptor for invasion by using *ex vivo* field isolates, and to identify band 3 ligand candidates by integrated transcriptome analysis. We used two complementary approaches (**Figure 1**). To investigate the potential role of the reticulocyte surface receptor band 3 in *P. vivax* invasion, we performed paired *ex vivo* invasion assays targeting band 3 in different ways. To identify band 3 ligand candidates, we used differential expression analysis of mRNA sequencing (mRNA-seq) data to link, for the first time, parasite phenotypes with gene expression profiles of the respective schizont-stage *P. vivax* isolates. Genes showing a strong association with invasion inhibition rates were considered candidate band 3 invasion ligands and were highly enriched in *PvTRAg* family members.

## Material and methods

### Ethics approval statement

Ethical approval for the collection and sequencing of *P. vivax* isolates from infected patients in Iquitos (Peru) was obtained from the Institute of Tropical Medicine Antwerp (ITM) Institutional Review Board (IRB; protocol 1345/19) and the ethics committee at the University Hospital of Antwerp (protocol B3002020000016) and Universidad Peruana Cayetano Heredia (UPCH; Lima, Peru) (protocol 101898). Cord blood collection in Iquitos was carried out under the same ethical approval from UPCH.

Ethical approval for the collection of umbilical cord blood and *P. vivax* isolates from infected patients in Madang (Papua New Guinea) was obtained from the ITM IRB (protocol 955/14), the ethics committee at the University Hospital of Antwerp (protocol B300201523588), the Papua New Guinea Institute for Medical Research IRB (protocol 1404), and the Papua New Guinea Medical Research Advisory Council (MRAC; protocol 14.07). Blood collection from patients with hemochromatosis in the ZNA Sint Erasmus and ZNA Stuivenberg hospitals (Antwerp, Belgium) was approved by the ITM IRB (protocol 946/14) and the ethics committee at the University Hospital of Antwerp (protocol B300201421346). Ethical approval for receptor genotyping was obtained from the respective local IRBs.

All adult participants provided written informed consent before enrolment. In the case of *P. vivax* blood collection from minors (12-18 years old) in Peru, written informed assent and consent were respectively obtained from minors and parents or guardians. For blood collection from minors (1-12 years old) in Papua New Guinea, written informed consent was obtained from a parent or guardian. The study was conducted according to the principles stated in the Declaration of Helsinki 2013 (World Medical Association, 2013).

### Sample collection

*P. vivax–*infected blood was collected from patients aged ≥ 12 years with acute *P. vivax* infection from the Peruvian city of Iquitos and neighboring communities and from children between aged 1-12 years from Madang in Papua New Guinea. All *P. vivax* cases were diagnosed by light microscopy. 10-20 mL samples of blood from patients with single *P. vivax* infections and parasite densities >0.1% and a gametocyte proportion <50% were collected in lithium-heparin tubes.

Reticulocytes were enriched from either umbilical cord blood or peripheral blood collected from patients with hemochromatosis. 10-100 mL of cord blood was collected in 50 mL falcon tubes containing 0.5 mL of heparin (5000U/mL) at hospital delivery wards in Madang, Papua New Guinea, and Iquitos, Peru. 450 mL of blood from hemochromatosis patients undergoing therapeutic phlebotomy was collected in Sepacell bags (Fresnius Kabi) at the ZNA Sint-Erasmus and ZNA Stuivenberg Hospitals in Antwerp, Belgium.

### Reticulocyte enrichment from umbilical cord blood and hemochromatosis patients

Reticulocytes were purified from umbilical cord blood or hemochromatosis blood samples within 48 hours of collection, following previously described protocols (Borlon et al., 2012). In brief, a blood sample aliquot was stored for future DNA extraction and genotyping. In the case of hemochromatosis blood, samples were typed for ABO blood type and Duffy phenotype (Fy) using standard serological methods (ABO/Rh Blood Typing Kit, Edulab and DiaMed-ID Micro Typing Systems, DiaMed, respectively). Leukocytes were removed using filters (Fresenius Kabi) and the reticulocytes were then concentrated by centrifugation (15 minutes at 2800 rpm, without a brake) through a Percoll gradient. The optimal Percoll gradient was selected for each sample after testing gradients ranging from 66%-74% on a small volume. The proportion of purified reticulocytes was calculated by light microscopy from a thin smear with new methylene blue staining (Reticulocyte stain, Sigma). Cells with two or more granules of reticulin were considered to be reticulocytes. Reticulocyte purity was in a range of 15%-90%, and only samples with a proportion of reticulocytes >25% were used in invasion assays. Freezing and thawing of reRBC samples was performed as previously described (Borlon et al., 2012).

### Detection of the Southeast Asian ovalocytosis (SAO) genotype

Cord blood collected at hospital delivery wards in Madang, Papua New Guinea, was morphologically checked for the SAO phenotype by microscopy. If >95% of the red blood cells had an oval shape, the sample was considered to be a potential SAO sample. Next, DNA was extracted from all cord blood samples with the FavorPrep™ 96-Well Genomic DNA Extraction Kit (Favorgen) following the manufacturer’s instructions. SAO genotype was determined using the PCR method described by Jarolim et al. (1991) (**Supplementary Table 1**).

### Genotyping of reticulocyte-enriched RBC samples

All reRBC samples used in the invasion assays were genotyped for common erythrocyte receptor polymorphisms by PCR (**Supplementary Table 2**). DNA was extracted from blood samples (umbilical cord blood or peripheral blood from hemochromatosis patients) using the QIAamp DNA mini kit (Qiagen), following the manufacturer’s instructions. Previously published methods with modifications were carried out to determine Duffy blood type (Yazdanbakhsh et al., 2000), SAO mutation (Jarolim et al., 1991), CR1 exon 22 polymorphism (Xiang et al., 1999), GYPC exon 3 deletion (Gerbich negative blood type) (Tavul et al., 2008), and alpha thalassemia– associated deletions (Chong et al., 2000). The primer sequences, amplification conditions, and used restriction enzymes are summarized in **Supplementary Table 1**.

### Flow cytometry of reticulocyte-enriched RBC samples

Abundance levels of band 3, GYPC, GYPA, transferrin, and DARC on reticulocyte surfaces were quantified on a FACSVerse 3-laser (BD) flow cytometer. Approximately 200,000 reRBCs were washed in cold PBS 4% BSA solution and incubated at 4°C with monoclonal antibodies (see **Table 1** for technical information). Secondary staining combining an anti-DARC primary antibody and a polyclonal secondary mouse antibody (conjugated with Alexa 488) was performed for DARC.

**Table 1:** Overview of antibodies used for flow cytometry and experimental conditions.

Target	Fluorophore	Brand	Catalog number	Antibody volume	Total reaction volume	Incubation time	Remarks
Transferrin	APC	BD Biosciences	551374	20 µL	75 µL or 100 µL	30 min	
Band 3	FITC	IBGRL	9439	0.4 µL	75 µL	30 min	Clone BRIC6, which was reported to fail to interact with SAO RBCs (Groves et al., 1993), and targets the extracellular loop between amino acids 545-567
GYPC	PE	IBGRL	9411	0.2 µL	75 µL	30 min	
GYPA	FITC	IBGRL	9415	0.5 µL	75 µL	30 min	
DARC	/	Absolute Antibody Ltd	Ab00893-1.1	0.3 µL	100 µL	40 min	
Mouse	Alexa 488	Abcam	ab150113	0.05 µL	100 µL	30 min	Secondary antibody to anti-DARC

Singlets were selected based on forward-scatter and side-scatter area-height plots. At least 50,000 transferrin-positive events (reticulocytes) were then gated to determine the median fluorescence intensity (MFI) of band 3, GYPC, GYPA and DARC (**Supplementary Figure 1**). For each reRBC sample used, a negative control without antibody was prepared to determine the threshold for distinguishing between band 3/GYPA/GYPC/DARC-positive and -negative events.

### *P. vivax* patient blood sample processing and parasite maturation

Blood samples from patients with *P. vivax* infection were processed within 6 hours of collection. Leukocytes and platelets were depleted using cellulose columns (Sriprawat et al., 2009). Then, 100µL of *P. vivax–*infected RBCs were stored at 50% hematocrit for future whole-genome sequencing (WGS). The remaining RBCs were cultured and matured to the schizont stage in McCoy’s 5A medium (Invitrogen) supplemented with 20% human serum and 0.2% glucose (Russell et al., 2011). The schizont-stage culture was then treated with trypsin, and centrifuged on a 45% Percoll gradient to concentrate mature schizonts (15 minutes at 2800 rpm, without a brake). The resulting pure schizonts were immediately used in invasion assays and in a subset of samples where >4 µL pure schizonts were available (listed in **Supplementary Table 3**); 1-2 µL of schizonts were stored for later mRNA-seq in 20 volumes of Trizol (Invitrogen) for Papua New Guinean samples (n=4, from non-SAO patients) or 300 µL RNAprotect (Qiagen) for Peruvian samples (n=13).

### *Ex vivo* invasion assays

*Ex vivo* invasion assays were performed as previously described (Russell et al., 2011). The concentrated mature schizonts were mixed with reRBCs at a ratio of 1:6 (0.5 µL schizonts and 3 µL reRBCs). Anti-band 3 pAb (Abcam, ab172129) or a PvTRAg38 peptide (Biomatik, custom synthesis of amino acid region 187-208) was then added, except for assays where SAO reRBCs were used. The parasites were cultivated in McCoy’s 5A medium (Gibco) supplemented with 20% human serum and 0.2% glucose to achieve a final volume of 150 µL per well. Invasion assays were set up in a pairwise fashion, where for each isolate a control well was prepared without antibody/peptide using the same reRBC sample. For the SAO assays, the control wells were prepared using non-SAO reRBCs. The reRBC samples used in paired assays are described in **Supplementary Tables 2, 3**. In addition, positive control wells for the pAb and peptide were prepared for some isolates, using mouse isotype IgG (Thermo Fisher Scientific, 31903) and BSA, respectively. To determine the antibody and peptide concentration used in the invasion assays, dose-response curves were performed in triplicate (**Supplementary Figure 2**).

Invasion was measured 24 hours post-invasion by counting the ring- and young trophozoite-stage parasitemia in at least 9000 RBCs, and dividing this number by the parasitemia in the paired control (**Supplementary Table 3**). An invasion assay was considered valid when the parasitemia in the control reRBC well was ≥0.5%. Approximately 50% of collected isolates died during the maturation process or could not reinvade *ex vivo* (control parasitemia <0.5%). Those samples were excluded from future transcriptome analysis.

### mRNA-sequencing

Pelleted RBCs from purified schizont-stage samples stored in RNAProtect (Peru) were disrupted by buffer RLT (Qiagen), after which they were loaded into an RNeasy Plus Mini column (Qiagen) and further processed following the manufacturer’s instructions. An on-column DNase treatment step was performed to eliminate any DNA remaining in the sample. RNA from purified schizont-stage samples stored in Trizol (Papua New Guinea) was isolated by chloroform phase separation. Debris was separated with a 2-minute spin at 2000 rpm and the supernatant incubated for 5 minutes at room temperature. Next, 0.2 volumes of chloroform were added, incubated for 3 minutes at room temperature, and centrifuged at 5000 rpm for 30 minutes at 4°C. The supernatant was recovered, an equal volume of 70% ethanol added, and the mixture was loaded into an RNeasy Plus Mini column, following the above-described steps.

Libraries were prepared with the Truseq stranded mRNA LT kit (Illumina) according to the manufacturer’s instructions, and sequenced on an Illumina HiSeq 1500 sequencer, resulting in 100 bp paired-end reads. Libraries with fewer than 10 x 10^6^ reads from isolates used for the SAO invasion assays were cleaned of adapter sequences using AMPure XP beads and resequenced on a HiSeq X ten with 150 bp paired-end reads. Fastq reads from both runs were pooled and processed together. The mRNA-seq data is available in the NCBI Sequence Read Archive (SRA) under BioProject PRJNA853709.

STAR v2.7.3 (Dobin et al., 2012) default settings were used to map the reads to the PVP01 reference genome (PlasmoDB version 46) and count the number of reads per gene. The same was done for the downloaded raw reads from mRNA-seq datasets previously published by Zhu et al. (2016) and Siegel et al. (2020).

On average, 86% of the reads obtained from the Peruvian isolates and 60% of those from the Papua New Guinean isolates mapped to the *P. vivax* PVP01 reference genome. Coinfection with *P. falciparum*, *P. malariae*, or *P. ovale* was ruled out using fastqscreen (Wingett and Andrews, 2018). mRNA-seq data from all isolates used in the differential expression analysis contained >25 million reads. For the transcriptional variation analysis, they contained >1 million reads (**Supplementary Figure 3**). One Papua New Guinean isolate was excluded from all analyses because the number of reads was <1 million. **Supplementary Figure 4** shows the number of detected genes for read subsets of different sizes from each sample, where saturation in the number of detected genes indicates sufficient sequencing depth. T36 and T54 mRNA-seq reads saturate at a lower level due to adapter carryover.

### Age estimation (hpi) of purified schizont-stage samples

Two *P. vivax* life-stage transcriptome series generated by Zhu et al. (2016) (smru1 and smru2) were used to estimate the age (hours post-invasion, hpi) of the purified schizont-stage samples from Peru and Papua New Guinea and the 4 schizont-stage isolates from Cambodia from Siegel et al. (2020). The smru1 dataset contains transcriptomes for 7 life-stage time points (6, 18, 24, 30, 36, 42 and 48 hpi), while the smru2 dataset contains transcriptomes for 8 time points (6, 12, 18, 24, 30, 36, 42 and 48 hpi). Smru1, smru2, and the 4 Cambodian isolates were processed following the approach described for mRNA-seq data for Peruvian and Papua New Guinean isolates, starting from the raw fastq data.

The mapped smru1 and smru2 reads were normalized to Transcripts Per Kilobase Million (TPM) using the TPMCalculator (NCBI). Genes with <10 TPM-normalized reads in total over the different time points were filtered out because of excessively low overall expression levels. Next, the TPM-normalized reads were regularized log transformed (rlog; DESEQ2) to remove heteroscedasticity. Genes that are not in the core genome (Pearson et al., 2016) were removed, since they would be expected to have a more variable expression pattern across isolates, making them less suitable for estimating schizont age.

Our mapped mRNA-seq data (Peru, Papua New Guinea) and the mapped public data set from Siegel et al. (2020) were TPM-normalized and regularized log transformed. Each sample was then Spearman-correlated with each time point from the smru1 and smru2 transcriptome series (**Supplementary Figure 5**). For the sequencing data for each isolate, the best correlating time point in smru1 and smru2 was selected as the estimated schizont age. Age estimations (hpi) using the smru1 and smru2 datasets were <6 hours apart for all the isolates, and the hpi average was selected as the final age estimate (**Supplementary Figure 6**). The transcriptomes of three isolates (Pv017, Pv030, Pv033) correlated with trophozoite stage, and these isolates were therefore excluded from the differential expression and variation analyses (but are included in the sequence repository).

### Transcriptional variation analysis

The mRNA-seq data from schizont-stage isolates from Peru (n=10), Papua New Guinea (n=3), and Cambodia (n=4, Siegel et al., 2020) were included in the transcriptional variation analysis (performed separately by country). Genes with a mean TPM <5 within a country were filtered out. Next, TPM-normalized reads were regularized log transformed (rlog; DESEQ2) to account for heteroscedasticity (**Supplementary Figure 7**). The standard deviation per gene was then calculated for each country.

### Differential expression analysis

**Figure 2:**
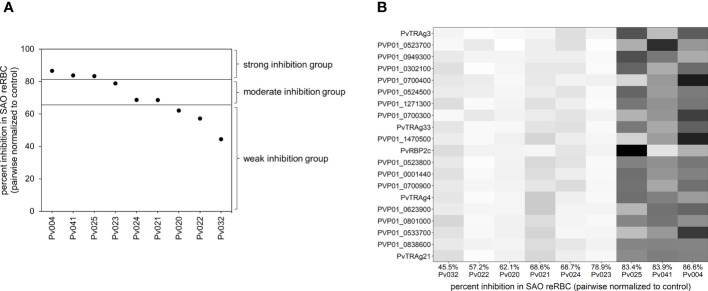
Visualisation of the differential expression analysis groups and outcome. **(A)** Plot showing the invasion inhibition level in SAO reRBCs for all nine isolates for which mRNA-seq data were obtained. From strong to weak inhibition, the isolates were split into three groups of three isolates. For four isolates (Pv004, Pv023, Pv024, Pv025) the SAO invasion inhibition level was repeated twice using SAO10 and SAO20 reRBCs. In those cases, the average invasion inhibition level was used and shown in the plot. **(B)** Heatmap showing, for each isolate, normalized expression values of the 20 genes in the candidate band 3 ligand list with the highest fold change. Isolates are ordered from weak (left) to strong (right) invasion inhibition levels in SAO reRBCs, and genes are ordered from high (top) to lower fold change (bottom). Darker fields indicate higher expression values.

To perform differential expression analysis, isolates were split in three groups (with three isolates per group) based on relative invasion inhibition in SAO invasion assays (**Figure 2A**) to account for the continuous nature of invasion inhibition data. Expression levels were compared between each group using DESEQ2 (Love et al., 2014). Upregulated genes identified in the strong vs weak, strong vs moderate, and moderate vs weak comparison were selected, as they potentially contain band 3 ligands. The Venn diagram in **Supplementary Figure 8** shows the number of up- and downregulated genes per comparison with overlapping genes. Because the weak and moderate groups contained very similar transcriptomes (only three differentially expressed genes), we continued with an intersection of the upregulated genes in the strong vs weak and strong vs moderate comparisons for further analysis. P-values were Benjamini-Hochberg corrected for multiple testing errors, and adjusted p-values <0.05 were considered significantly differentially expressed.

### GO analysis

Curated and computed GO annotations for the PVP01 reference genome (version 46) were accessed from PlasmoDB (date: 11/01/2021). The TopGO package in R was used to find significantly overrepresented GO terms in the candidate band 3 ligand list. The weight01 algorithm was used to take the hierarchy of the terms in the GO graph into account. The Fisher’s exact test was used to identify enriched GO terms.

### Co-expression analysis

TPM-normalized and regularized log transformed reads from the Peruvian schizont-stage isolates (n=10) were used to construct a co-expression network of *PvTRAg* genes. Transcriptional patterns of single genes across the different isolates were Spearman-correlated to each other. Visualization in an undirected, correlation coefficient–weighted network was prepared using the R package igraph. Only edges with a significant (p<0.05) Spearman correlation coefficient >0.9 or <-0.9 were retained, and nodes without connection were removed.

### Whole-genome sequencing

DNA from the leukocyte-depleted Peruvian blood samples Pv004, Pv020, Pv021, Pv022, Pv024, Pv025, Pv032, and Pv041 was extracted using the QIAamp DNA mini kit, following the manufacturer’s instructions. Libraries were prepared with the Nextera DNA Flex kit (Illumina), and sequenced on a HiSeq X ten machine (Illumina), resulting in 150 bp paired-end reads. The raw mRNA-seq data is available in the NCBI Sequence Read Archive (SRA) under BioProject PRJNA853729.

Reads were mapped to the PVP01 reference genome (PlasmoDB version 46) using bwa v0.7.17 with default settings, then coordinate sorted with samtools v1.9 and duplicate marked with picard v2.22.0. Variants were then called using the GATK (v4.1.4.1) HaploTypeCaller with GATK-recommended settings (-ERC GVCF); the resulting gvcf files for the eight isolates were combined and converted to a vcf file with GATK. The vcf was split in a SNP and an indel vcf, after which filter fields were defined according to the GATK golden standard (for the SNP vcf: QD<2.0, QUAL<30.0, SOR>3.0, FS>60.0, MQ<40.0, MQRankSum<-12.5, ReadPosRankSum<-8.0; for the indel vcf: QD<2.0, QUAL<30.0, FS>200.0, ReadPosRankSum<-20.0). Only variants that passed the filters described, were binary, and had a coverage ≥10 in all eight samples were retained in the vcf files.

### Maximum likelihood phylogenetic tree

To construct a maximum likelihood tree using the WGS data, only core genome SNPs were retained in the filtered SNP vcf file (Pearson et al., 2016). The resulting vcf was converted to a fasta-format multiple sequence alignment (MSA) using vcf2phylip (Ortiz, 2019). Based on this MSA, a maximum likelihood tree was constructed with RaxML (v8.2.11) (Stamatakis, 2014), using 100 rapid bootstrapping inferences followed by a search for the best-scoring maximum likelihood tree (settings: -m GTRGAMMA -f a -x 1 -N 100). Evolutionary distances were computed using the generalized time-reversible (GTR) substitution matrix, and the rate variation among sites was modelled with a gamma distribution. The Newick format tree was visualized in FigTree (v1.4.4).

### Statistical analysis

The Wilcoxon signed-rank tests were performed in R v3.6.1 (R Core Team, 2019); 95% confidence intervals were calculated in Excel, and are based on the sample standard deviation and Student’s t-distribution to account for small sample sizes.

## Results

### The band 3 receptor mediates an invasion pathway into reticulocytes

**Figure 3:**
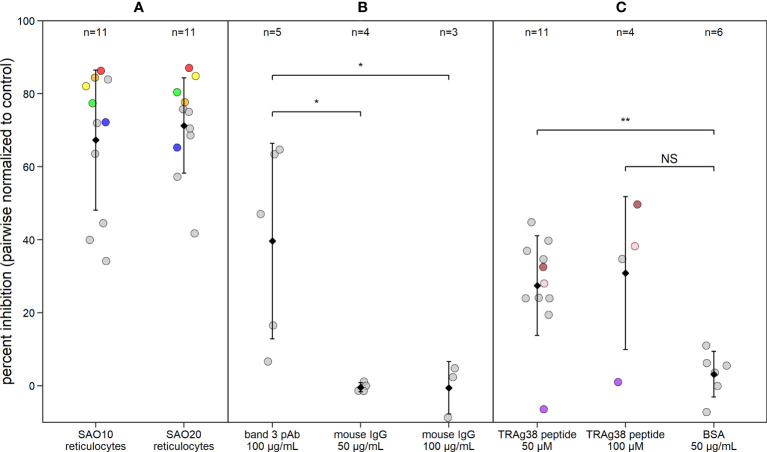
*P. vivax* reticulocyte invasion efficiency is dependent on band 3 receptor availability. Dot plot showing *P. vivax* reticulocyte invasion inhibition in invasion assays using **(A)** two SAO reRBC samples (SAO10 and SAO20), **(B)** a polyclonal antibody (pAb) against band 3 and **(C)** a PvTRAg38 peptide (amino acid region 187-208). Percent inhibition for each *P. vivax* isolate is pairwise-normalized to the invasion observed in the control reRBCs (non-SAO reRBCs, in absence of antibodies or peptides). Dots represent isolates. Black diamonds represent the mean percentage of invasion inhibition, with whiskers showing the standard deviation. Isolates used twice in the presence of different SAO reRBCs or at a different TRAg38 peptide concentration are shown in the same color. Mouse IgG 50 µg/mL and 100 µg/mL = band 3 pAb positive control, BSA = PvTRAg38 peptide positive control. Wilcoxon signed rank test was performed to test for significant differences between the invasion assays and their positive controls. **=p<0.01, *=p<0.05, NS, not significant.

To investigate the potential role of band 3 in *P. vivax* invasion, isolates collected from patients in Iquitos, Peru, were matured to the schizont stage for use in invasion assays. SAO reticulocytes with mutant band 3 provide an important experimental model for such assays and were enriched from SAO genotype cord blood from Papua New Guinea. We performed a pairwise comparison of *P. vivax* invasion in SAO reticulocyte-enriched RBC (reRBC) samples (SAO10 and SAO20) and non-SAO control reRBC samples with non-mutated band 3 (**Figure 3A**) (n=22). *P. vivax* invasion was 67% ± 13% and 71% ± 9% lower in SAO10 and SAO20 reRBCs (mean invasion inhibition ± 95% confidence interval) than in non-SAO reRBCs, respectively. These results demonstrate strong *P. vivax* invasion inhibition in SAO reticulocytes, hinting at a role for band 3 as a receptor in reticulocyte invasion.

To further investigate the band 3 invasion pathway in reticulocytes, we performed invasion assays in the presence of anti-band 3 polyclonal antibody (pAb), to block the potential parasite binding site to band 3. P*. vivax* invasion was 40% ± 33% (mean ± confidence interval) lower in reRBCs incubated with anti-band 3 pAb than in control reRBCs without antibody (**Figure 3B**; n=5), further supporting the hypothesis that band 3 plays a role in invasion. Nonetheless, considerable variation was observed in invasion inhibition levels, both when using SAO reRBCs and anti-band 3 pAb, suggesting that band 3–related *P. vivax* invasion of reRBCs varies across isolates.

**Figure 4:**
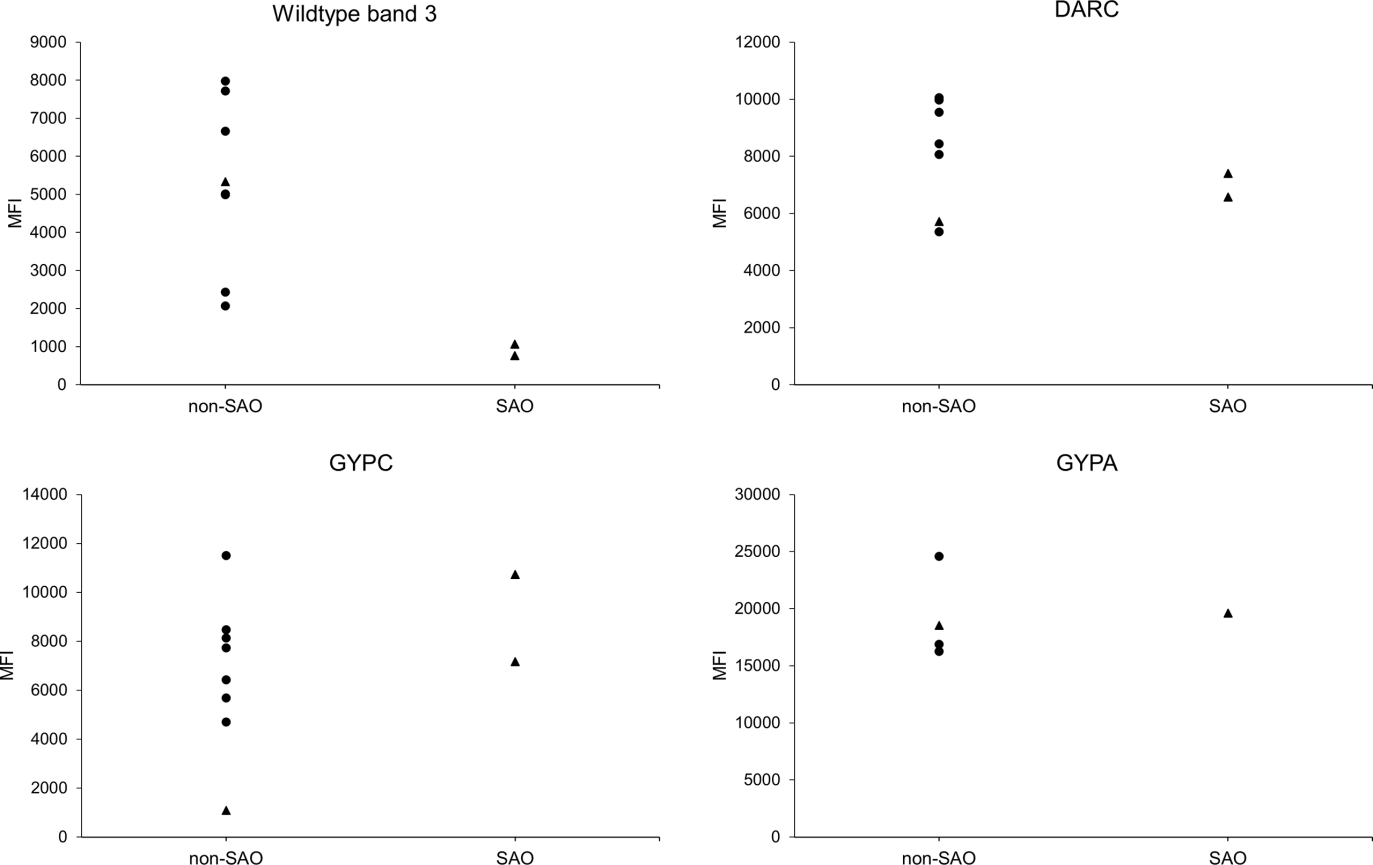
Receptor expression on SAO and non-SAO reticulocytes as measured by flow cytometry. Surface abundance of band 3, DARC, GYPC, and GYPA receptors on SAO and non-SAO reticulocytes (only CD71+ RBCs were measured) is expressed as median fluorescent intensity (MFI). The SAO reticulocytes used were SAO10 and SAO20, which were also used in the invasion assays (only SAO20 for GYPA). The dots indicate reRBC samples from hemochromatosis blood collected in Belgium, and triangles reRBC samples from cord blood collected in Papua New Guinea. reRBCs were incubated with commercial fluorescently tagged antibodies, using primary (band 3, GYPC, GYPA) or secondary (DARC) binding. The anti-band 3 antibody used was reported to specifically target wildtype band 3 (Groves et al., 1993). The non-SAO reRBC sample from Papua New Guinea (triangle) has a homozygous GYPC exon 3 deletion, resulting in a low MFI for GYPC compared with the other reRBC samples. No statistical tests could be performed due to the small number of SAO reRBCs.

The use of a SAO reRBC model to study band 3 invasion can be potentially confounded by the presence of other RBC polymorphisms or an altered availability of other receptors due to the SAO deletion. To control for this, we checked for the presence of highly prevalent RBC polymorphisms in Papua New Guinea (alpha thalassemia, CR1 exon 22 polymorphism, and GYPC exon 3 deletion) in the reRBC samples used in this study. We observed the presence of variants in both the control and SAO reRBCs (**Supplementary Table 2**). To test whether these genetic variants might have affected *P. vivax* invasion of SAO reRBCs, we compared invasion inhibition levels in SAO10 and SAO20 reRBCs infected with the same isolate (n=5), and observed no significant differences (paired Wilcoxon signed-rank test p=0.81) (**Supplementary Figure 9**). This indicates that these additional receptor polymorphisms most likely did not affect level of invasion. To ensure that the lower levels of invasion observed in SAO reRBCs were not related to the reduced availability of other receptors or membrane proteins due to band 3–related conformational changes in macrocomplexes, we measured the abundance of antigen receptors by flow cytometry. SAO10 and SAO20 reRBC samples had similar levels of DARC, GYPC, and GYPA to non-SAO reRBC samples, but decreased levels of wildtype band 3, as the BRIC6 antibody does not recognize band 3 in the presence of the SLC4A1Δ27 deletion (Groves et al., 1993) (**Figure 4**). Flow cytometry analysis also confirmed earlier findings that band 3 is equally present in normocytes and reticulocytes (**Supplementary Figure 10**) (Malleret et al., 2017). Due to the limited number of SAO reRBCs available for this study, our findings cannot be generalized to all SAO RBCs.

### A PvTRAg38 peptide competes with the band 3 ligand for binding

To investigate the role of the PvTRAg38-band 3 interaction in *P. vivax* invasion, reRBCs were incubated with the PvTRAg38 peptide that was previously shown to bind to band 3 (amino acid 187-208) (Alam et al., 2015; Alam et al., 2016a) at a concentration of 50 µM and 100 µM. *P. vivax* invasion was 27% ± 9% and 31% ± 33% (mean ± 95% confidence interval) lower in reRBCs incubated with 50 µM (n=11) and 100 µM of peptide (n=4), than in control reRBCs, respectively (**Figure 3C**). These results indicate that the PvTRAg38 peptide competes to some extent with the endogenous PvTRAg38 ligand for binding to the band 3 receptor for most isolates, suggesting in turn that the PvTRAg38-band 3 interaction was involved in reticulocyte invasion in most but not all the *P. vivax* isolates studied in this experiment.

### Transcriptional variation of *PvTRAg* and *PvRBP* genes is high between schizont isolates

We observed considerable variation in invasion inhibition levels across *P. vivax* isolates. The ability of *P. vivax* parasites to invade reticulocytes using different receptor-pathways (Kanjee et al., 2020; Malleret et al., 2021) may reflect different ligand expression profiles that result in specific or redundant receptor binding preferences. To test this hypothesis, transcriptome sequencing (mRNA-seq) was performed on 13 Peruvian *P. vivax* field isolates that were matured *ex vivo*; of these, 10 were estimated to be schizont stage based on correlations with life stage–specific transcriptomes generated by Zhu et al. (2016) (**Supplementary Figure 6A**).

**Figure 5:**
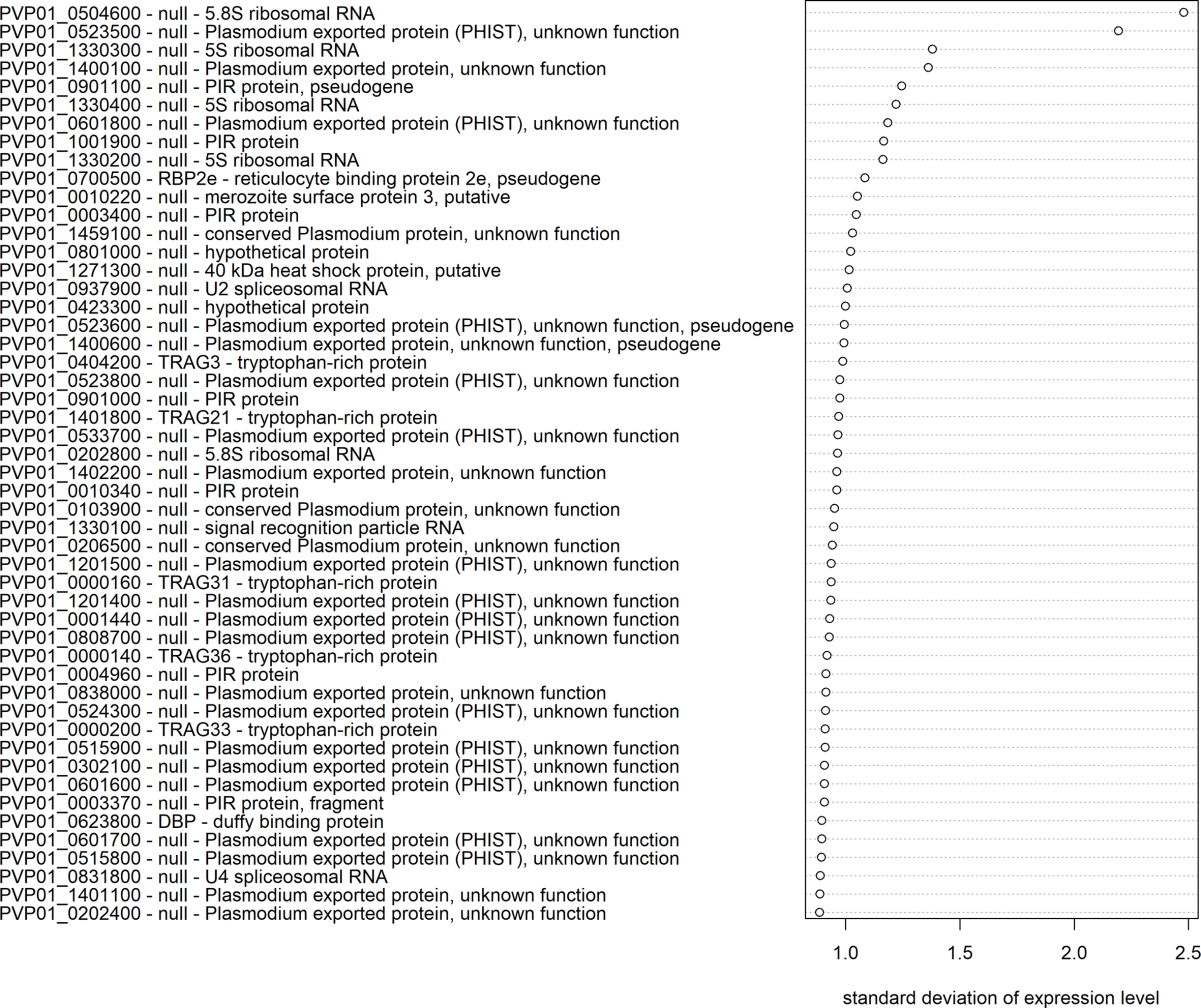
Transcriptional variation in *P. vivax* schizont stages from Peru. The 50 genes with the highest transcriptional variation (standard deviation) in Peruvian schizont stage isolates (n=10), with their gene ID, gene name, and product description. Expression levels were normalized and made homoscedastic, after which standard deviation was calculated per gene and used as a measure of variability.

The extent of transcriptional variation was investigated using mRNA-seq data for all confirmed schizont-stage isolates. The transcript data were first normalized and made homoscedastic, after which the standard deviation was calculated as a measure of variation between isolates. Three multigene families, *PHIST* (30%), *PIR* (14%), and *PvTRAg* (10%), were most predominantly represented in the top 50 most variably expressed genes (**Figure 5**). *PHIST* and *PvTRAg* were also significantly enriched (Fisher’s exact test p≥0.001). Several of the top 50 most variably expressed genes are (potentially) linked to invasion, namely, five *PvTRAg* genes, *PvDBP*, and one *PvRBP* gene (*PvRBP2e*, a pseudogene).

**Figure 6:**
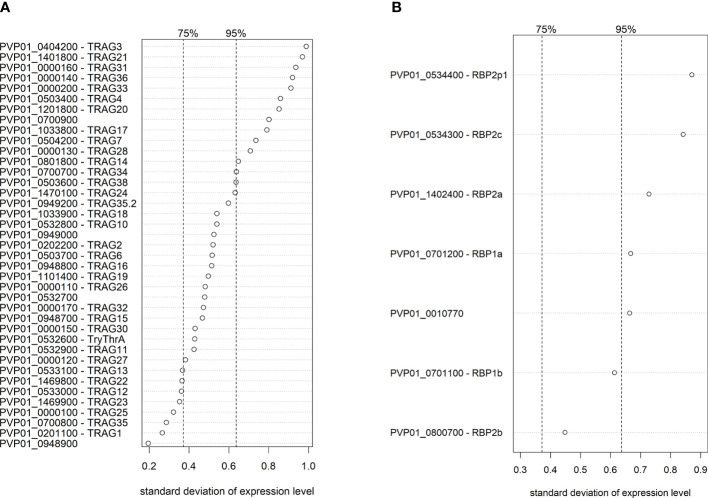
Ranking of transcriptional variation (standard deviation) of the *PvTRAg* **(A)** and *PvRBP* **(B)** family members in Peruvian schizont isolates (n=10). Dashed lines indicate the 75% and 95% quantile, indicating that on the right of these lines are respectively the 25% and 5% most variable genes of the genome. Expression levels were normalized and made homoscedastic, after which standard deviation was calculated per gene and used as a measure of variability. The *PvTRAg* gene PVP01_0000210 was excluded from the analysis due to <5 normalized transcript counts.

When examining the *PvTRAg* and *PvRBP* families, which both contain potential ligands for invasion (Zeeshan et al., 2014; Gruszczyk et al., 2018; Chan et al., 2019; Malleret et al., 2021), 13/40 *PvTRAg* genes and 5/7 *PvRBP* genes were in the top 5% of genes with the highest transcriptional variation across Peruvian isolates. *PvTRAg3* had the highest expression variability of all *PvTRAg* genes, followed by *PvTRAg21*, *31* and *36*, which have been previously shown to bind *in vitro* to band 3, basigin, and an unknown receptor  (Zeeshan et al., 2014) (**Figure 6A**). *PvRBP2p1* was the most variable *PvRBP* member, while, interestingly, the transferrin ligand *PvRBP2b* was the least variable of all *PvRBP*s (**Figure 6B**).

To uncover potential spatial diversity in *PvTRAg* and *PvRBP* expression between strains from different geographic regions, we repeated the same mRNA-seq analysis with 3 schizont-stage isolates from Papua New Guinea, and 4 publicly available schizont-stage isolates from Cambodia (Siegel et al., 2020), processed using identical methods as those used for the Peruvian isolates. *PvTRAg* and *PvRBP* expression variability (i.e., ranking of genes) varied by geographic region, and different family members were highly expressed in Peru compared with Papua New Guinea and Cambodia (**Supplementary Figures 11A–D**). Although the sample size was small for Papua New Guinean and Cambodian isolates, the geographic differences observed might have implications for the generalizability of our findings for the Peruvian isolates.

### Schizont transcriptomes reveal band 3 ligand candidates

We hypothesized that transcriptional variation in the *PvRBP* and *PvTRAg* candidate ligand families might be associated with the variation in band 3 usage observed in the invasion assays. Accordingly, correlations between levels of invasion inhibition and the transcriptional profiles of different isolates could reveal potential band 3 ligand(s). Observation of strong invasion inhibition (**Figures 3A** **, B**), for instance, would suggest that a given isolate is strongly reliant on band 3 as a receptor for reticulocyte invasion and probably expresses relatively high levels of one or more band 3 receptor ligands. Weak inhibition, however, would indicate that the isolate is not dependent on the availability of the band 3 receptor for invasion (it may use an alternative receptor) and probably expresses low levels of band 3 ligand(s).

To investigate this hypothesis without prior assumptions of PvTRAg or PvRBP involvement, we performed differential expression analysis on isolates from Peru for which there were sufficient schizonts to perform both SAO invasion assays and mRNA sequencing (n=9).

First, we defined the comparison groups for the differential expression analysis. Unlike classic differential expression analysis, which compares transcriptomes between two groups constructed using discrete data, we opted to define 3 groups that reflect the continuous nature of invasion inhibition in SAO reRBCs. The nine parasite isolates were thus divided into strong, moderate, and weak invasion inhibition groups according to their SAO invasion inhibition level (**Figure 2A**).

Second, to find potential band 3 ligands, we performed differential expression analysis to compare isolate transcriptomes between the strong and the moderate and weak inhibition groups. Overall, 254 genes (3.7% of all genes) were significantly upregulated in the strong inhibition group for both comparisons (strong vs weak and strong vs moderate) and were accordingly identified as candidate ligands for the band 3 receptor, hereafter named “candidate band 3 ligand list” (**Supplementary Table 4** shows the final candidate band 3 ligand list [A] and between-group comparisons [B-D]). The MA and volcano plots are shown in **Supplementary Figure 12**. Based on the Gene Ontology (GO) cellular compartment annotation, this list can be narrowed down to 209 band 3 ligand candidates, as proteins not located in the rhoptries, micronemes, or merozoite membrane will most likely not be ligands (**Supplementary Table 4A**).

The candidate band 3 ligand list contains many invasion-related genes, including *PvRBP2a, RPB2c*, *PvDBP*, *PvDBP2*, *PvCyRPA*, and 15 *PvTRAg*s. The top 20 genes with the highest fold changes (expression increase) between the strong and the weak or moderate inhibition groups are shown in **Figure 2B**. Our findings are supported by GO analysis, which indicated a significant enrichment of host cell surface receptor binding proteins in the candidate band 3 ligand list (Fisher’s exact test p<0.001). The presence of 2/7 *PvRBP*s and 15/40 *PvTRA*gs among the candidates is more than expected by chance (Fisher’s exact test p<0.05 and p<0.001, respectively). *PvTRAg* genes, in particular, are strongly overrepresented in the candidate band 3 ligand list (Fisher’s exact test p<0.001), indicating a possible association with band 3–mediated invasion. *PvTRAg38*, which showed variable inhibition levels in the invasion assays (**Figure 3C**), was also identified as a candidate band 3 ligand. However, we were unable to confirm the expected link between level of inhibition observed during blockage with the PvTRAg38 peptide and level of *PvTRAg38* expression (higher in isolates with stronger PvTRAg38 peptide inhibition) (**Supplementary Figure 13**). This might reflect redundancy within the PvTRAg family, with other PvTRAg ligands possibly governing the interaction with band 3 or another receptor when the PvTRAg38 binding site is blocked (Zeeshan et al., 2014).

To check that the previously selected differentially expressed genes were not the consequence of genetic bias between the comparison groups, we performed whole-genome sequencing (WGS) for eight of the nine mRNA-sequenced isolates and constructed a maximum likelihood tree. The resulting clades did not match our three comparison groups (**Supplementary Figure 14**), indicating that mutations in the isolate genomes were randomly distributed between the groups and thus unlikely to affect the results of the differential expression analysis. Similarly, modest differences in schizont age (**Supplementary Figure 15**) and reRBC polymorphisms (**Supplementary Tables 2, 3**) were randomly distributed between the comparison groups.

### Co-expression in the *PvTRAg* gene family suggests functional redundancy

**Figure 7:**
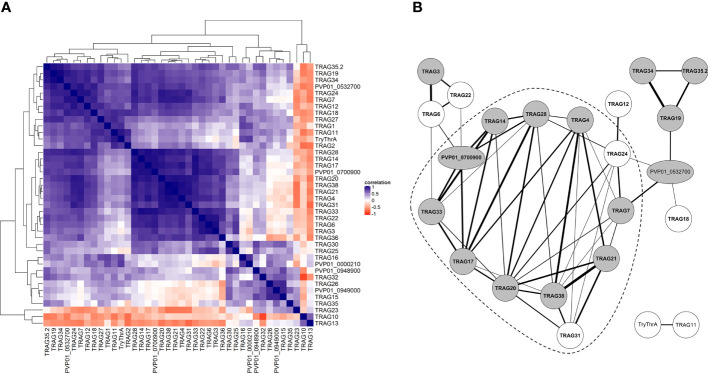
Co-expression analysis of *PvTRAg* genes during schizont stage. **(A)** Clustered correlation matrix of expression patterns of all *PvTRAg* genes during schizont stage in 10 Peruvian isolates. Level of correlation (Spearman correlation coefficient) is shown by a red-blue scale, where dark blue indicates strong correlation or co-expression and dark red strong anticorrelation or opposite expression pattern. For *PvTRAg* genes without a product name, the geneID is shown. **(B)** Co-expression network where nodes represent genes and edges connect co-expressed genes, based on the correlation of *PvTRAg* schizont transcriptomes in 10 Peruvian isolates. Only edges with a significant Spearman correlation coefficient >0.9 are included. Edge width corresponds to correlation level. Gray nodes indicate *PvTRAg*’s that are present in the candidate band 3 ligand list (constructed by differential expression analysis), bold font represents *PvTRAg*’s expressed during schizont stage according to the time series from Zhu et al. (2016). The dashed line surrounds the module of highly interconnected *PvTRAg* genes (≥3 edges with other *PvTRAg* genes from the same module). For *PvTRAg* genes without a product name, the geneID is shown.

To gain greater insights into the regulation of the *PvTRAg* family, we constructed a co-expression matrix with schizont-stage transcriptome data from isolates from Peru (n=10) (**Figure 7A**). Overall, 23/40 *PvTRAg* genes showed strong co-expression (Spearman’s correlation coefficient >0.9, p<0.05) with at least one other *PvTRAg* gene. When these strongly co-expressed genes were linked in a network (**Figure 7B**), this revealed a highly interconnected group or module (≥3 connections with other module members) comprising 12 *PvTRAg* genes, 10 of which were in the candidate band 3 ligand list. *PvTRAg* genes in this module might be regulated through the same mechanism and share a similar function.

## Discussion

In this study, we combined *ex vivo* invasion assays and transcriptome sequencing to uncover a band 3–mediated invasion pathway in *P. vivax* and potential band 3 ligands. Functional invasion assays based on short-term culture of *P. vivax* parasites are the experimental approach that most closely mimics the natural invasion process, and are therefore an optimal tool for studying invasion in the absence of continuous culture and laboratory strains. We performed 22 successful invasion assays with SAO reRBCs and 17 P*. vivax* isolates, an unprecedentedly high number in the *P. vivax* literature (Barnwell et al., 1989; Grimberg et al., 2007; Gruszczyk et al., 2018; Carias et al., 2019; Knuepfer et al., 2019; Prajapati et al., 2019; Kanjee et al., 2020; Malleret et al., 2021). We also performed five invasion assays with anti-band 3 pAb and 15 with a PvTRAg38 peptide. Invasion was inhibited in the presence of SAO10 and SAO20 reRBCs, an anti-band 3 pAb, and a PvTRAg38 peptide, showing that band 3 is a receptor for *P. vivax* invasion and that PvTRAg38 is an invasion ligand.

Considerable differences in invasion inhibition levels were observed across the *P. vivax* isolates in each of the three invasion assays (SAO vs non-SAO reRBCs, anti-band 3 pAb, and PvTRAg38 peptide). High levels of variation across isolates have also been reported in invasion assays targeting DARC (Kanjee et al., 2020), transferrin (Gruszczyk et al., 2018; Kanjee et al., 2020), CD98 (Malleret et al., 2021) and basigin (combination of newly acquired data and results from Knuepfer et al., 2019; **Supplementary Figure 16**). It has been shown that the main source of variation in invasion assays is not the reRBC samples used but the parasite isolates (Noulin et al., 2012; Kanjee et al., 2020). We also observed no significant differences in invasion inhibition levels in SAO10 and SAO20 assays when the same isolate was used. The large invasion inhibition variations observed overall in our study indicate the existence of at least one alternative pathway to the band 3-mediated pathway. Some isolates rely heavily on the availability of band 3 for successful reRBC invasion, while for others, invasion is unaffected or only modestly affected when band 3 availability in the reRBC membrane is low or blocked. Similar results have been reported for *P. falciparum* laboratory strains, with most parasite lines strongly dependent on band 3, and just a few using an alternative RBC invasion pathway  (Cortés et al., 2004). Band 3 does not contribute to the *P. vivax* tropism for reticulocytes, opposed to CD71 and CD98 (Malleret et al., 2017; Gruszczyk et al., 2018; Malleret et al., 2021), and isolates show varying dependence on band 3 presence. Therefore, band 3 is most likely a co-receptor involved in the invasion process alongside other known receptors DARC, CD71 and CD98. We cannot conclude how band 3 is exactly embedded in the multistep process of invasion. The interdependency between band 3 and other receptors, and extent of alternative - potentially DARC-independent - pathways should be further investigated by blocking different receptor combinations.

In addition to the between-isolate variability described above, we also observed different mean levels of invasion inhibition in the three invasion assay setups (SAO, anti-band 3 pAb, and PvTRAg38 peptide). There are several possible explanations for the difference observed between SAO and pAb invasion inhibition (67%-71% vs 40%). First, incomplete blocking of the band 3 binding site may have occurred if the concentration of anti-band 3 pAb did not fully saturate the highly abundant band 3 receptor on the surface of the reticulocytes, as the dose response curve suggests. Second, availability of other potential receptors not targeted in the flow cytometry assays may have been affected by the SAO mutation, although this possibility was mitigated by targeting proteins from both the 4.1R and band 3 complexes. Third, an increase in RBC tension may have further reduced invasion into SAO reRBCs, as previously described for *P. falciparum* infection in Dantu genotype RBCs (Kariuki et al., 2020). However, some *P. falciparum* lines invade SAO RBCs efficiently (Cortés et al., 2004), and high-density *P. falciparum* infections have been reported for SAO individuals (Allen et al., 1999), which indicates that the increased rigidity of SAO cells alone cannot explain the observed inhibition. However, we cannot exclude increased tension as a contributing factor, alongside with the changed band 3 availability or conformation.

The high variation and lower levels of invasion inhibition observed for the PvTRAg38 peptide compared with SAO and anti-band 3 pAb (27%-31% vs 67%-71% and 40%) may be due to the presence of redundant ligands or cooperative activity from other ligands that bind to different band 3 epitopes. These additional ligands may be PvTRAg36, PvTRAg22, or PvGAMA, which have all been shown to bind to band 3 *in vitro* (Zeeshan et al., 2014; Alam et al., 2016b; Lu et al., 2022), or any one of the other candidate band 3 ligands identified in the differential expression analysis (e.g., other PvTRAgs). It should also be noted that the PvTRAg38 peptide may be less efficient in binding to the band 3 receptor than the endogenous, natively folded PvTRAg38 ligand.

We hypothesize that transcriptional variation underlies the variation in band 3 receptor usage across isolates. This hypothesis is supported by the high variability observed in the expression of multigene families and known or potential ligands. Overall, members of the multigene families *PvTRAg*, *PIR*, and *PHIST* were among the most variably expressed genes across 10 Peruvian schizont transcriptomes, supporting previous findings for the Cambodian *P. vivax* schizont isolates  (Siegel et al., 2020). *PHIST* genes are annotated as “exported protein with unknown function”, and their function indeed remains largely unknown in *P. vivax*. The *P. falciparum PIR* family is involved in antigenic variation, immune evasion, invasion, sequestration, and rosetting  (Niang et al., 2009; Niang et al., 2014; Goel et al., 2015). In *P. vivax*, the *PIR* family shows transcriptional variation across life cycle stages (Sa et al., 2020; Little et al., 2021), suggesting that different PIR members may be involved in different functions. Apart from the potential PvTRAg invasion ligands, the DARC-ligand *PvDBP* also showed high transcriptional variation across isolates, possibly explaining the previously observed variation in DARC-mediated *P. vivax* invasion efficiency (Kanjee et al., 2020). The *PvRBP* family also showed high variability, with five gene members among the 5% most variable genes. Even though *PvRBP2b*, the ligand to transferrin, was the least variably expressed gene within the *PvRBP* family, variation in *PvRBP2b* expression might explain the previously observed variation in invasion inhibition when transferrin-mediated invasion was blocked  (Kanjee et al., 2020).

The high transcriptional variation in schizont-stage multigene families could be caused by epigenetic variations. In *P. falciparum*, ligand multigene families located in subtelomeric regions, such as *eba*, *rhoph1/clag*, and *PfRh*, are typically epigenetically regulated (Cortés et al., 2007), and so is the *P. yoelii* invasion family *Py235* (Preiser et al., 1999). Epigenetic regulation of clonally expressed genes involved in invasion provides a selective advantage through host immune evasion or lower vulnerability to receptor changes on the host erythrocyte. Epigenetic regulation in *P. vivax* is poorly studied, and single-cell approaches would be needed to elucidate silenced or activated gene expression patterns of individual parasites.

We hypothesized that the variable transcriptional patterns observed in our study could provide information on band 3 ligand(s) when linked to functional invasion assays. Previous studies have shown a link between *P. falciparum* invasion profiles and ligand upregulation, such as the association between the sialic acid–independent CR1 pathway and the upregulation of the PfRh4 ligand (Stubbs et al., 2005; Gaur et al., 2006). In our study, differential expression analysis identified 254 genes that were highly expressed in isolates with high invasion inhibition levels in SAO reRBCs (dependency on band 3 availability for invasion) and lowly expressed in isolates with low invasion inhibition levels (non-dependency on band 3 availability). These genes were included in what we called the “candidate band 3 ligand list”. It is inherent to differential expression analysis that genes unrelated to band 3–mediated invasion will also be in the candidate band 3 ligand list as well, because of similar regulatory mechanisms involved in gene expression. However, this analysis narrows down the band 3 ligand options and is an important resource for further validation studies.

Of the 15 *PvTRAg* genes identified as potential band 3 ligands by transcriptional analysis, recombinant PvTRAg21, 38, 19, and 35.2 have been shown to bind to mature RBCs (Zeeshan et al., 2014) and PvTRAg38 to band 3 *in vitro* (Zeeshan et al., 2014; Alam et al., 2015; Alam et al., 2016a; Alam et al., 2016b). In our study, the PvTRAg38 peptide reduced invasion by 27%-31% *ex vivo*. While recombinant PvTRAg22 and PvTRAg36 have also been shown to bind to band 3 *in vitro*, they were not identified as ligand candidates in the differential expression analysis. PvGAMA on the other hand, which also binds to band 3 *in vitro*, was present in the candidate band 3 ligand list (Lu et al., 2022).

Enrichment analysis, a commonly used tool to help interpret differentially expressed genes and extract patterns of interest, revealed strong overrepresentation of *PvTRAg* genes in the candidate band 3 ligand list, suggesting a possible role for the *PvTRAg* genes in band 3–mediated invasion. A previous comparison of Aotus (DARC-mediated invasion) and Saimiri (DARC-independent invasion) monkey transcriptomes showed that *PvTRAg* genes are more abundantly expressed in parasites infecting Saimiri monkeys, indicating that their proteins may act as ligands in DARC-alternative invasion pathways (Gunalan et al., 2019). We observed high levels of *PvTRAg* co-expression, especially for genes expressed during the schizont stage and genes in the candidate band 3 ligand list. *PvTRAg* co-expression suggests shared regulatory mechanisms and therefore, potential shared function, such as being ligands involved in invasion, either through binding to band 3 or other receptors, such as basigin and the unknown receptor B (Zeeshan et al., 2014).

We used a differential expression approach to identify band 3 ligand candidates in which transcriptomes were grouped by phenotypic characterization according to the invasion capacity of the isolates. A similar approach could be used to investigate both invasion pathways involving alternative RBC receptors, and other phenotypes, such as drug resistance and parasite virulence. Considering that continuous culture is still lacking for *P. vivax*, transcriptome data might provide new insights into *P. vivax* biological processes. *P. vivax* transcriptome studies are scarce and have mainly focused on transcriptional changes throughout the erythrocytic cycle (Bozdech et al., 2008; Zhu et al., 2016; Rangel et al., 2020; Sa et al., 2020). Transcriptional variation within specific life cycle stages and potential geographic differences are understudied, with just 4 Cambodian schizont-stage isolates analyzed to date  (Siegel et al., 2020). The 13 new *P. vivax* transcriptomes generated in this study thus are an important contribution to current understanding of schizont transcriptomes and transcriptional variability between isolates, while the combination of transcriptomic and phenotypic data is unique to *P. vivax* isolates. A concerted effort by the *P. vivax* community to integrate ‘omics and phenotypic data and to study samples from various geographic locations would open opportunities to gain a deeper understanding of *P. vivax* biological processes and their complexity.

In conclusion, we have demonstrated that band 3 functions as a receptor in *P. vivax* invasion and that PvTRAg38 is at least one of its ligands. Dependence on PvTRAg38 for invasion varies across isolates, with expression data supporting a redundant ligand function for several *PvTRAg* genes. In our search for band 3 ligands, we have, for the first time, coupled *P. vivax* phenotypes – in this case band 3 usage for invasion – to transcriptomes from the same isolates. Based on this analysis, *PvTRAg* genes emerged as the most likely band 3 ligand candidates. Future studies should seek to further unravel their complex regulation and function through single-cell sequencing, transgenic *P. knowlesi* or *P. cynomolgi* models, or additional *P. vivax* invasion assays targeting PvTRAgs and combinations thereof. How band 3 relates to other receptors and invasion pathways could be further investigated by blocking different receptor combinations.

## Data availability statement

The datasets presented in this study can be found in online repositories. The names of the repository/repositories and accession number(s) can be found below:

https://www.ncbi.nlm.nih.gov/bioproject/PRJNA853709

https://www.ncbi.nlm.nih.gov/bioproject/PRJNA853729

## Ethics statement

The studies involving human participants were reviewed and approved by the Institute of Tropical Medicine Antwerp (ITM) Institutional Review Board (IRB), the Ethics committee of the University Hospital of Antwerp, the Ethics committee of the Universidad Peruana Cayetano Heredia, the Papua New Guinea Institute for Medical Research IRB, and the Papua New Guinea Medical Research Advisory Council (MRAC). Written informed consent to participate in this study was provided by the participants or their legal guardian/next of kin.

## Author contributions

AR-U conceived the project, whereafter SP, KD, AR-U, and JK designed the experimental work. AR-U supervised the overall study, KL supervised the bioinformatic work, and LR, JK, ML, and DG supervised the field collections and experimental work performed on-site. SAO and Non-SAO reRBCs were purified and characterized by SP and BK. KD led the bioinformatic analysis and interpreted the results with significant contributions from BC. SP and KD carried out the invasion assays with contributions from EV and BK. JK carried out the RNA-Seq library preparation of the schizont-stage isolates, KD prepared the WGS library. KD performed flow cytometry of the reRBC, while SP, BK, and KD performed PCR genotyping of reRBC samples. KD, AR-U, KL, and BC interpreted the results. KD, AR-U, and BC wrote the first draft of the manuscript. All authors read and approved the final manuscript.

## Funding

This work was supported by the Department of Economy, Science and Innovation in Flanders (SOFI to ARU), and the Research Foundation Flanders (1S48419N scholarship to KDM, V417919N and V414020N travel grants to KDM, K207216N and V440515N travel grants to SKP). The computational resources and services used in this work were provided by the HPC core facility CalcUA of the Universiteit Antwerpen, and VSC (Flemish Supercomputer Center), funded by the Research Foundation Flanders (FWO) and the Flemish Government.

## Acknowledgments

We would like to thank the patients, clinical staff, microscopists, and field staff from health centers and hospitals in and around Iquitos (Peru) and Madang (Papua New Guinea) for their cooperation and contribution to the collection of *P. vivax* isolates. In particular, dr. John Bolgna, Head of Obstetrics and Gynacology at Modilon Hospital, for his leadership and support of the project. We also would like to acknowledge the participants that donated cord blood and the nurses from the hospitals in Madang and Iquitos that helped with this collection. EV acknowledges the financial support of Proyecto Concytec – Banco Mundial “Mejoramiento y Ampliación de los Servicios del Sistema Nacional de Ciencia Tecnología e Innovación Tecnológica” 8682-PE, through its executing unit ProCiencia (08-2018-FONDECYT/BM-Programas de Doctorados en Áreas Estratégicas y Generales). We are grateful to Jan Van Den Abbeele for his thorough revision of the manuscript, Pieter Meysman for his insights on the bioinformatic analyses, and Diego Segura for his help with the sample collection and invasion assays in Iquitos, Peru.

## Conflict of interest

The authors declare that the research was conducted in the absence of any commercial or financial relationships that could be construed as a potential conflict of interest.

## Publisher’s note

All claims expressed in this article are solely those of the authors and do not necessarily represent those of their affiliated organizations, or those of the publisher, the editors and the reviewers. Any product that may be evaluated in this article, or claim that may be made by its manufacturer, is not guaranteed or endorsed by the publisher.

## Supplementary material

Supplementary Table 1 PCR programs, primer sequences, and restriction enzymes used for each of the different reRBC polymorphism PCRs carried out. Protocols were modified from Yazdanbakhsh et al. (2000) for the Duffy blood type, Jarolim et al. (1991) for the SAO genotype, Xiang et al. (1999) for the CR1 exon 22 polymorphism, Tavul et al. (2008) for the Gerbich blood type, and Chong et al. (2000) for the alpha thalassemia–associated deletions.

Supplementary Table 2 List of reRBC samples used for the invasion assays and flow cytometry experiments, with ABO blood type, reticulocyte purity, and polymorphisms for each reRBC sample.

Supplementary Table 3 Overview of invasion assays, with isolate used, reRBC sample, and percent inhibition compared with the paired control.

Supplementary Table 4 Table showing the candidate band 3 ligand list **(A)** as well as comparisons between all inhibition groups: strong vs weak inhibition group **(B)**, strong vs moderate **(C)**, and moderate vs weak **(D)**, with their log2 transformed fold changes, baseMean (average of normalized count values for all samples), lfcSE (standard error estimate for the log2 fold change), stat (value of the test statistic for the gene or transcript), p-value, and Benjamini-Hochberg adjusted p-value (padj). The cellular compartment GO terms shown for the candidate band 3 ligand list **(A)** are both computed and curated. Log2 transformed fold changes and Benjamini-Hochberg adjusted p-values (padj) are the mean of the values obtained for the strong vs weak and strong vs moderate group comparisons.

## Abbreviations

Complement Receptor 1, CR1; DARC, Duffy, Duffy antigen receptor for chemokines; GO, Gene Ontology; GYPA, Glycophorin A; GYPC, Glycophorin C; PvGAMA, GPI-anchored micronemal antigen; hpi, Hours post-invasion; TfR1, CD71, Human transferrin receptor 1; MFI, Median fluorescence intensity; MSA, Multiple sequence alignment; PvDBP, *P. vivax* Duffy binding protein; PvEBP, *P. vivax* erythrocyte binding protein; PvRBP, *P. vivax* reticulocyte binding protein; PvTRAg, *P. vivax* tryptophan-rich antigen; pAb, Polyclonal antibody; RBC, Red blood cell; rlog, Regularized log transformation; reRBCs, Reticulocyte-enriched red blood cells; SAO, Southeast Asian ovalocytosis; TPM, Transcripts per kilobase million; WGS, Whole-genome sequencing.
