# Supplementary material for: Band 3–mediated Plasmodium vivax invasion is associated with transcriptional variation in PvTRAg genes
Source: Front Cell Infect Microbiol. 2022 Sep 30;12:1011692. doi: 10.3389/fcimb.2022.1011692 (PMC9563252; doi:10.3389/fcimb.2022.1011692)
Supplement: Supplementary file 5 [file DataSheet_1.pdf]

Supplementary Material

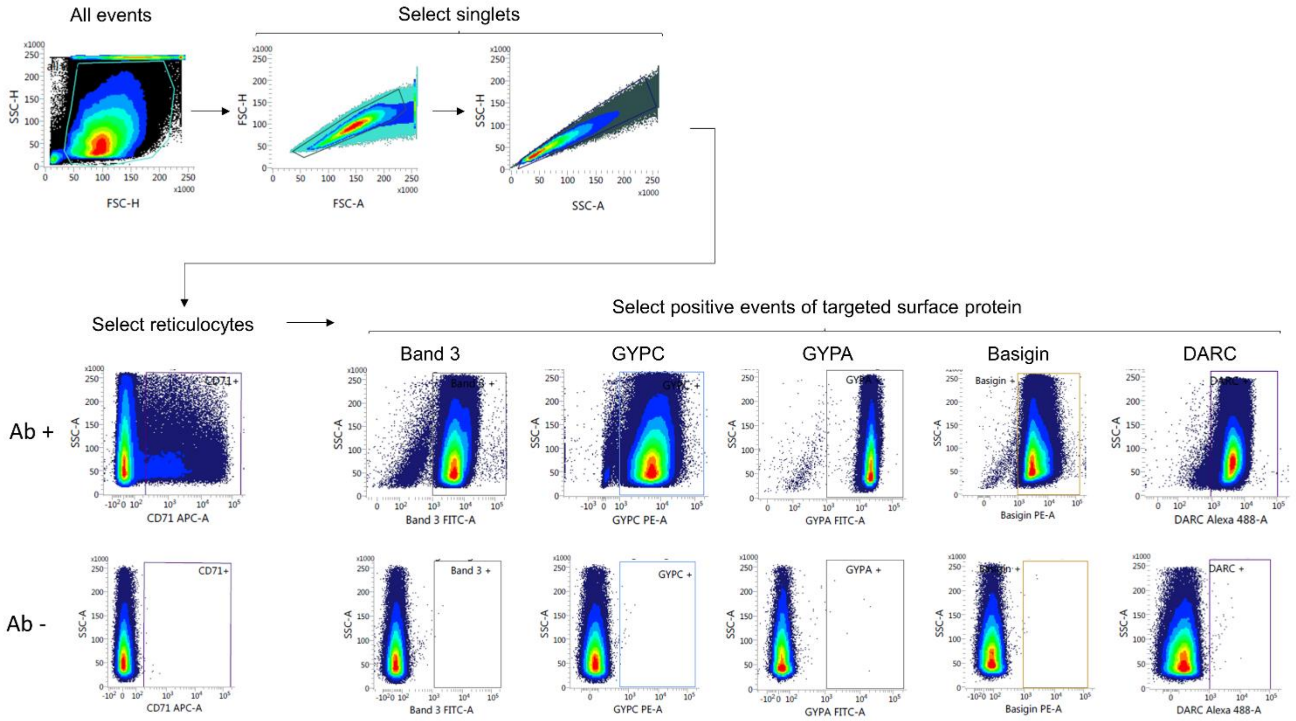

Supplementary Figure 1. Gating strategy used for quantification of surface abundance levels of band 3, GYPC, GYPA, basigin, and DARC on reticulocytes. Data are shown for the reRBC sample HCR39. Ab+: antibodies added, Ab-: negative control without added antibodies (same reRBC sample).

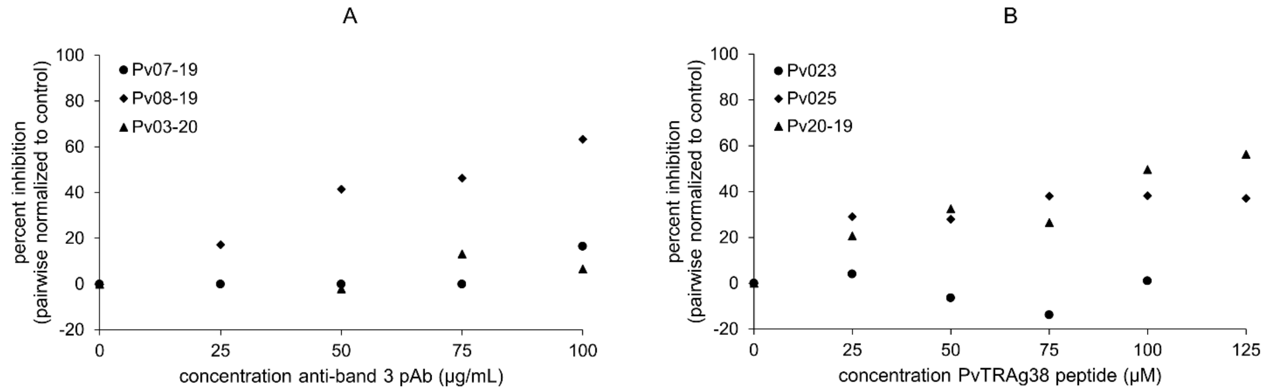

Supplementary Figure 2. Dose response curves (n=3) to determine the inhibitor concentration to use in invasion assays. Invasion assays were set up using increasing concentrations of **(A)** anti-band 3 polyclonal antibody and **(B)** TRAg38 peptide (amino acid region 187-208), each done in triplicate. Invasion inhibition levels are pairwise normalized to the inhibition observed in the control (same reRBC sample without the presence of antibodies or peptides) using the same *P. vivax* isolate.

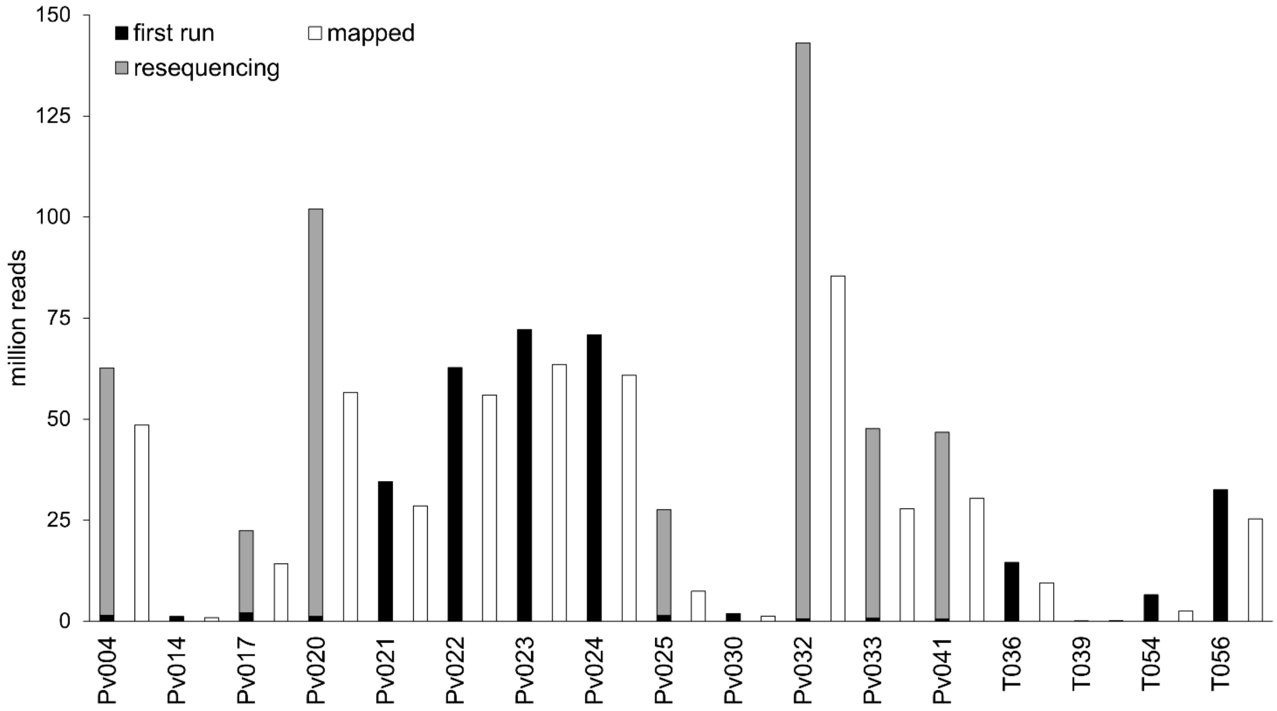

Supplementary Figure 3. Number of raw RNA-Seq reads per sample for the first run (gray) and its resequencing (black), and number of reads mapping to the PVP01 reference genome (white bars).

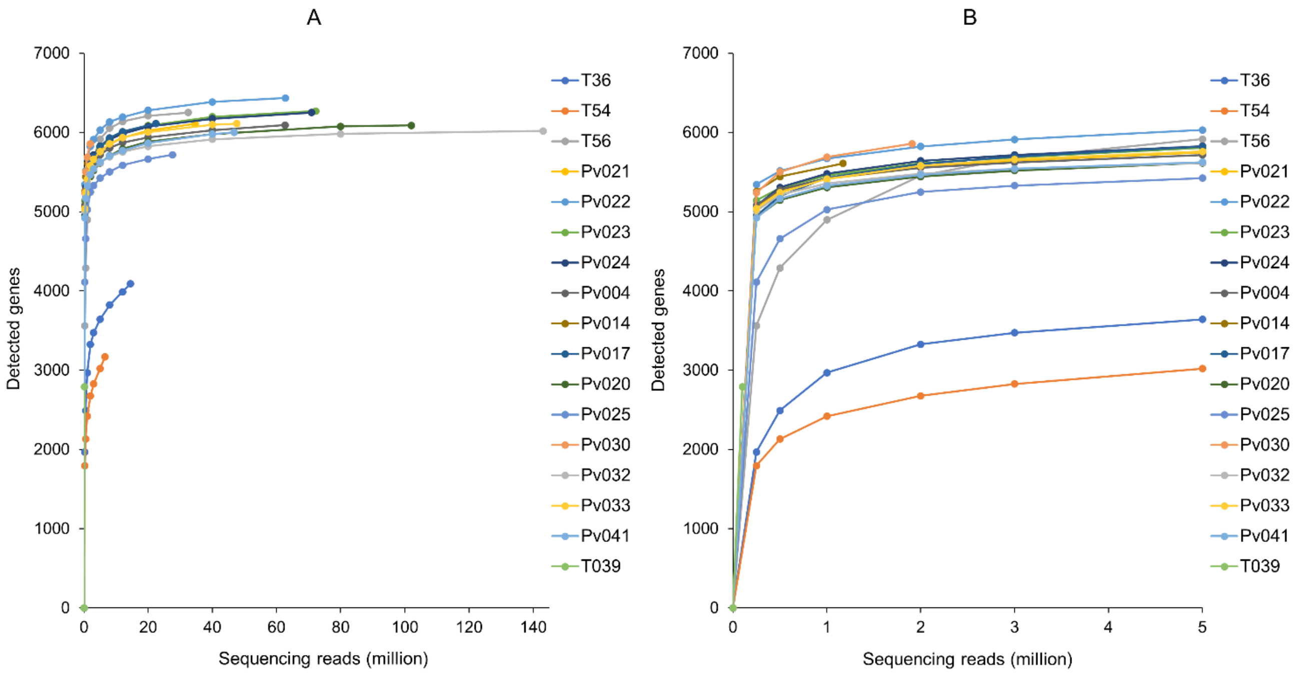

Supplementary Figure 4. Number of expressed genes detected in random subsets of raw reads (forward and reverse reads were sampled as pairs) for the Peruvian isolates **(A)** and the same plot zoomed in on the region of 0-5 million reads **(B)**. Saturation of the number of detected genes indicates sufficiently deep sequencing.

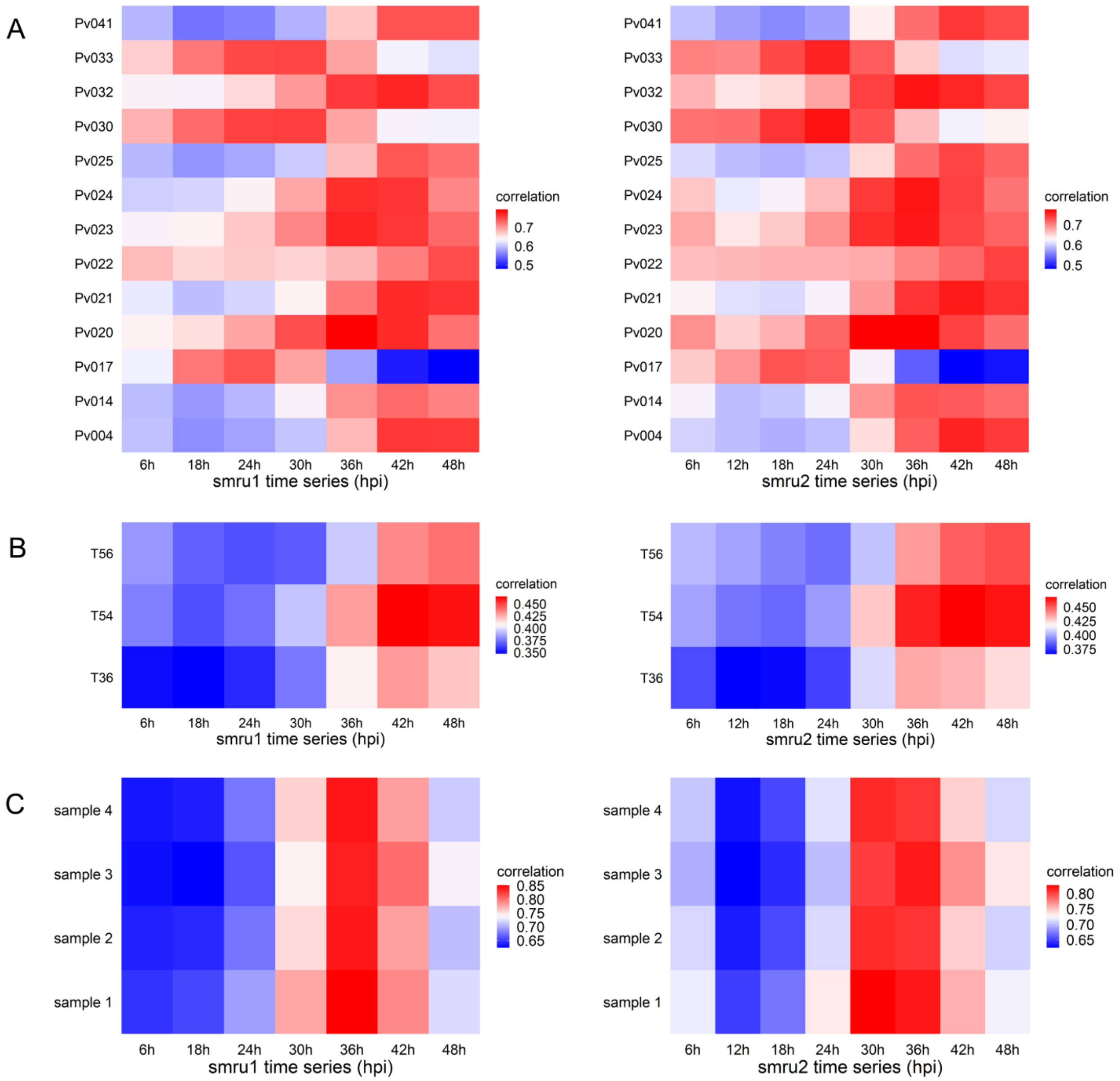

Supplementary Figure 5. Heatmaps showing the Spearman correlation of each sample with both time series of Zhu et al. (2016) (smru1 and smru2). **(A)** Peruvian samples, **(B)** Papua New Guinean samples, **(C)** Cambodian samples of Siegel et al. (2020). x-axis indicates hours post-invasion.

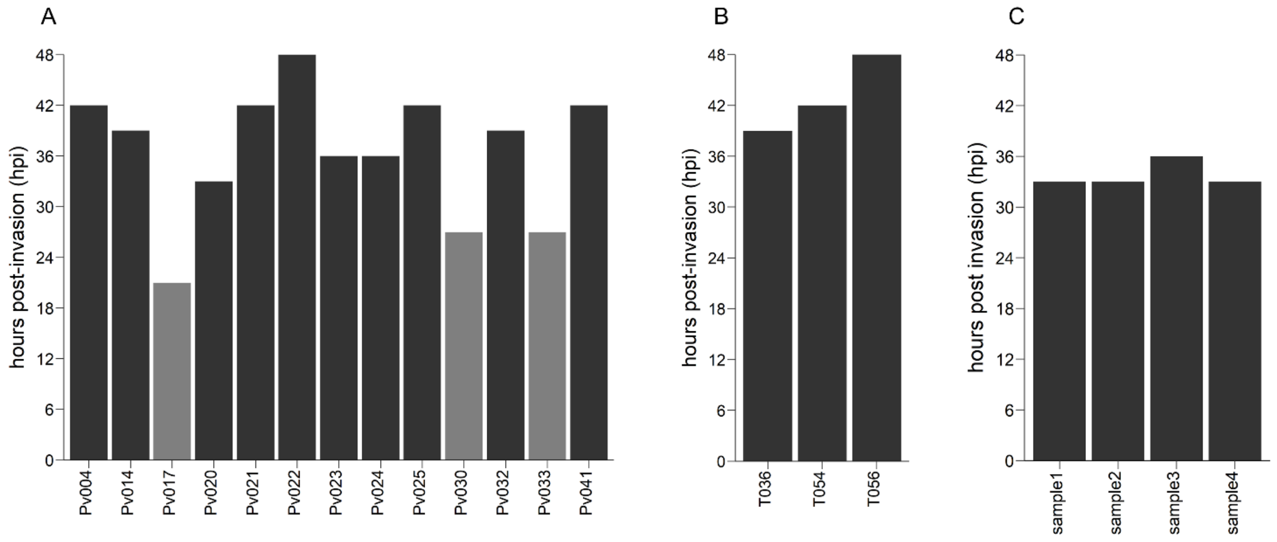

Supplementary Figure 6. Bar plot showing the estimated parasite age (hours post-invasion; hpi) at the moment of harvesting for mRNA-Seq of in-house isolates from Peru **(A)** and Papua New Guinea **(B)**, and the publicly available dataset from Siegel et al. (2020) **(C)**. Isolates excluded from the analysis (no schizont stage) are colored in a lighter shade of gray. Transcriptomes were Spearman-correlated to the time point–specific transcriptomes generated by Zhu et al. (2016), and the highest correlating time point was considered the estimated parasite age (hpi) of the RNA-Seq sample.

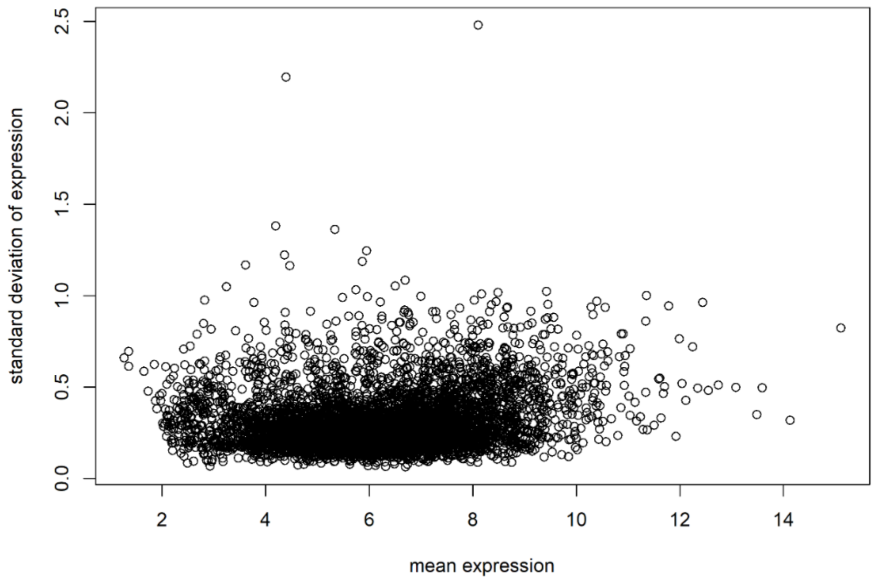

Supplementary Figure 7. Plot showing standard deviation of gene expression levels (open circles) from Peruvian samples (n=10) as a function of mean gene expression. Gene expression levels are TPM normalized and variance stabilized (rlog function DESEQ2).

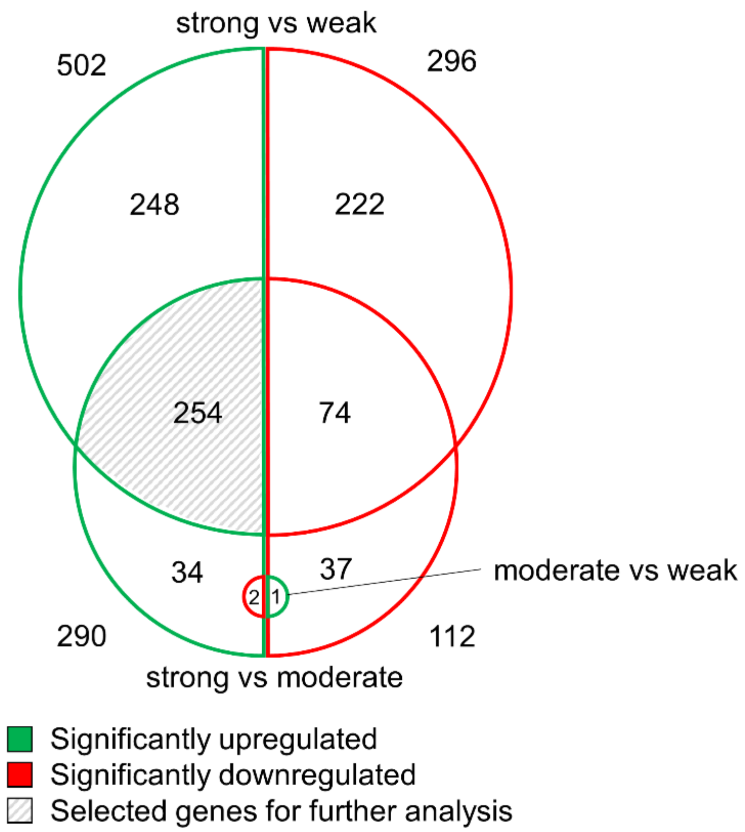

Supplementary Figure 8. Venn diagram showing upregulated and downregulated genes for all pairwise comparisons between the strong, weak, and moderate inhibition groups. The shaded area contains candidate band 3 ligand genes (resulting from an overlap between strong vs weak and strong vs moderate upregulated genes), and those genes were selected for further analysis.

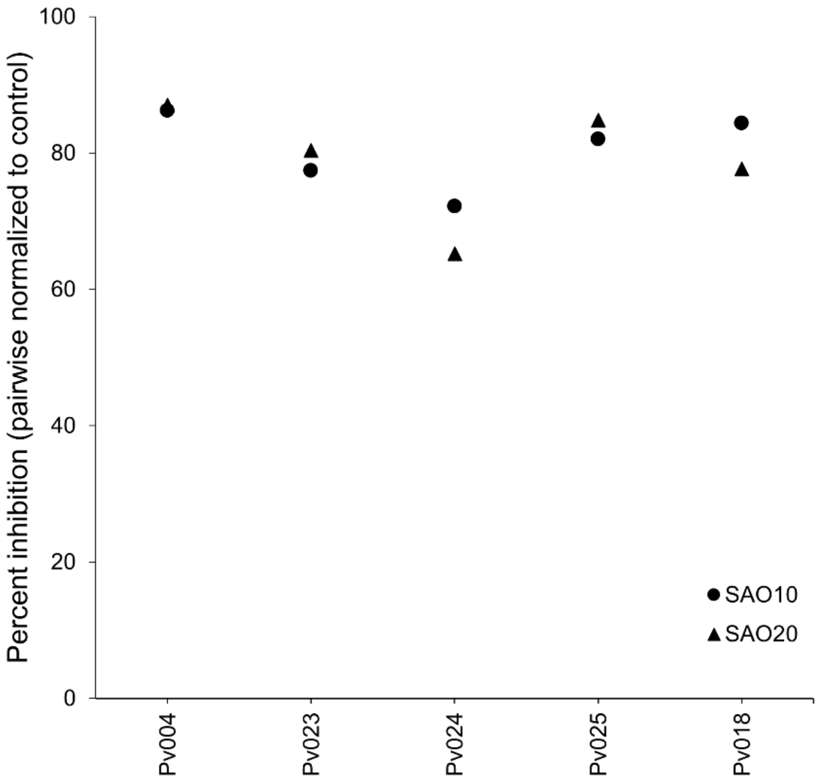

Supplementary Figure 9. No significant differences were observed for invasion inhibition for the two SAO reRBC samples (SAO10 and SAO20). For five isolates, the SAO invasion assays were performed twice with SAO10 and SAO20 (the two reRBC samples used in all SAO invasion assays). Invasion inhibition levels were pairwise normalized to the inhibition observed in the control (non-SAO reRBCs) using the same *P. vivax* isolate.

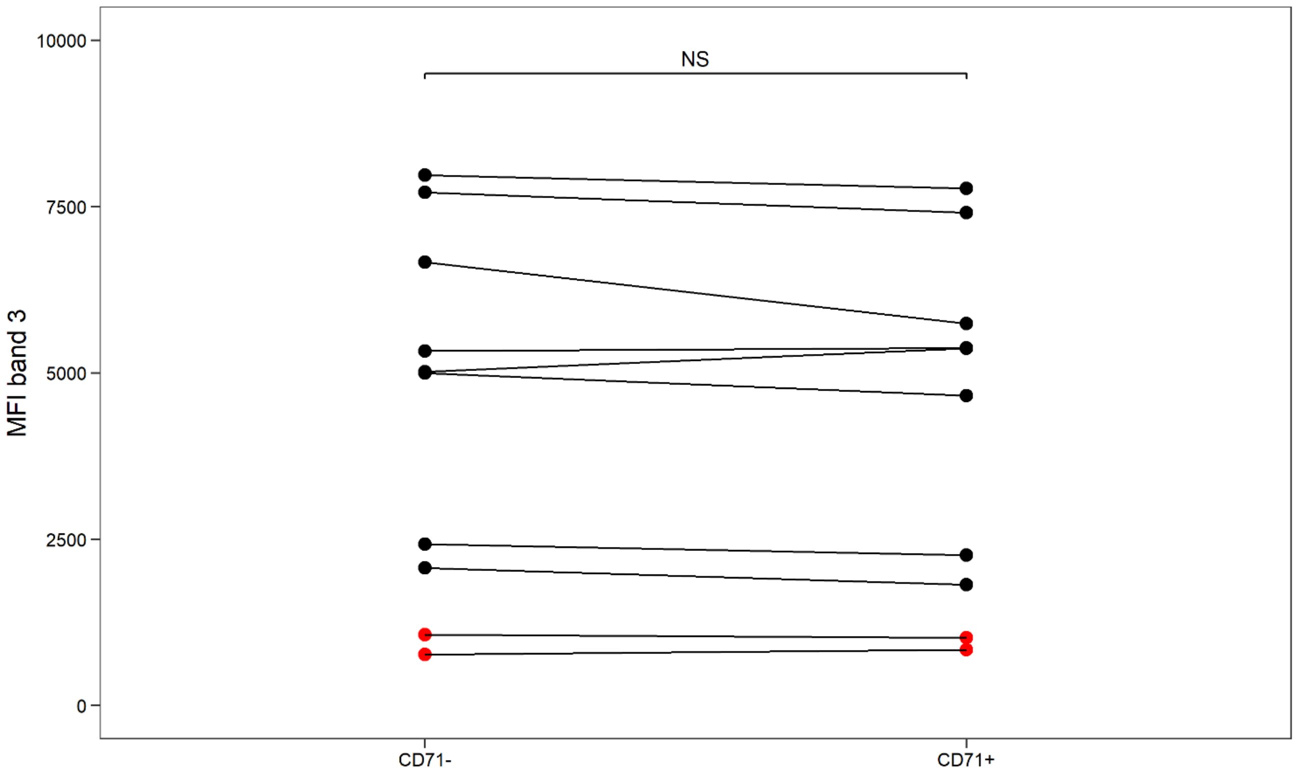

Supplementary Figure 10. Band 3 surface abundance on CD71- (mature RBCs) and CD71- (reticulocytes) RBCs, as measured by flow cytometry. Same reRBC samples are connected with a black line. Red dots indicate SAO RBCs (SAO10 and SAO20). Surface abundance is expressed as median fluorescent intensity (MFI). The used anti-band 3 antibody was reported to specifically target wildtype band 3 (Groves et al. 1993). A pairwise t-test revealed no significant (NS) difference in band 3 presence between the CD71- and CD71+ group.

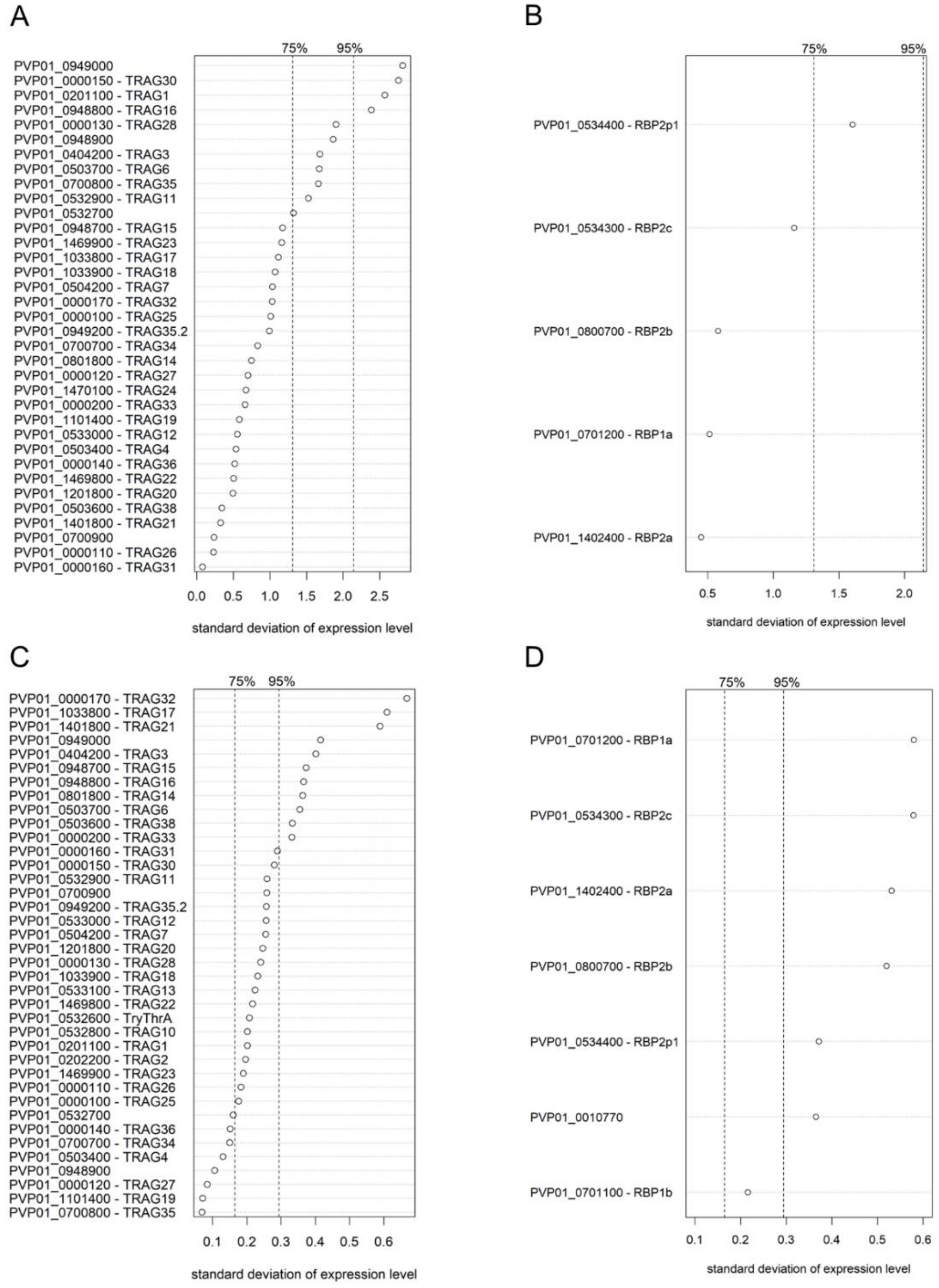

Supplementary Figure 11. Ranking of transcriptional variation (standard deviation) of *PvTRAg* and *PvRBP* family members in Papua New Guinean schizont samples (n=3) **(A, B)** and publicly available Cambodian schizont samples (n=4) (Siegel et al. 2020) **(C, D)**. Dashed lines indicate the 75% and 95% quantile, indicating that on the right of these lines are, respectively, the 25% and 5% most variable genes of the genome. Expression levels were normalized and made homoscedastic, after which standard deviation was calculated per gene and used as a measure of variability. Some *PvTRAg* and *PvRBP* genes were excluded from the analysis as they had <5 normalized transcript counts (A: *PvTRAg* genes PVP01_0000210, *TryThrA*, *PvTRAg2*, *PvTRAg10*, and *PvTRAg13*; B: *PvRBP* genes *PvRBP1b* and PVP01_0010770; C: *PvTRAg* genes *PvTRAg24*, and PVP01_0000210).

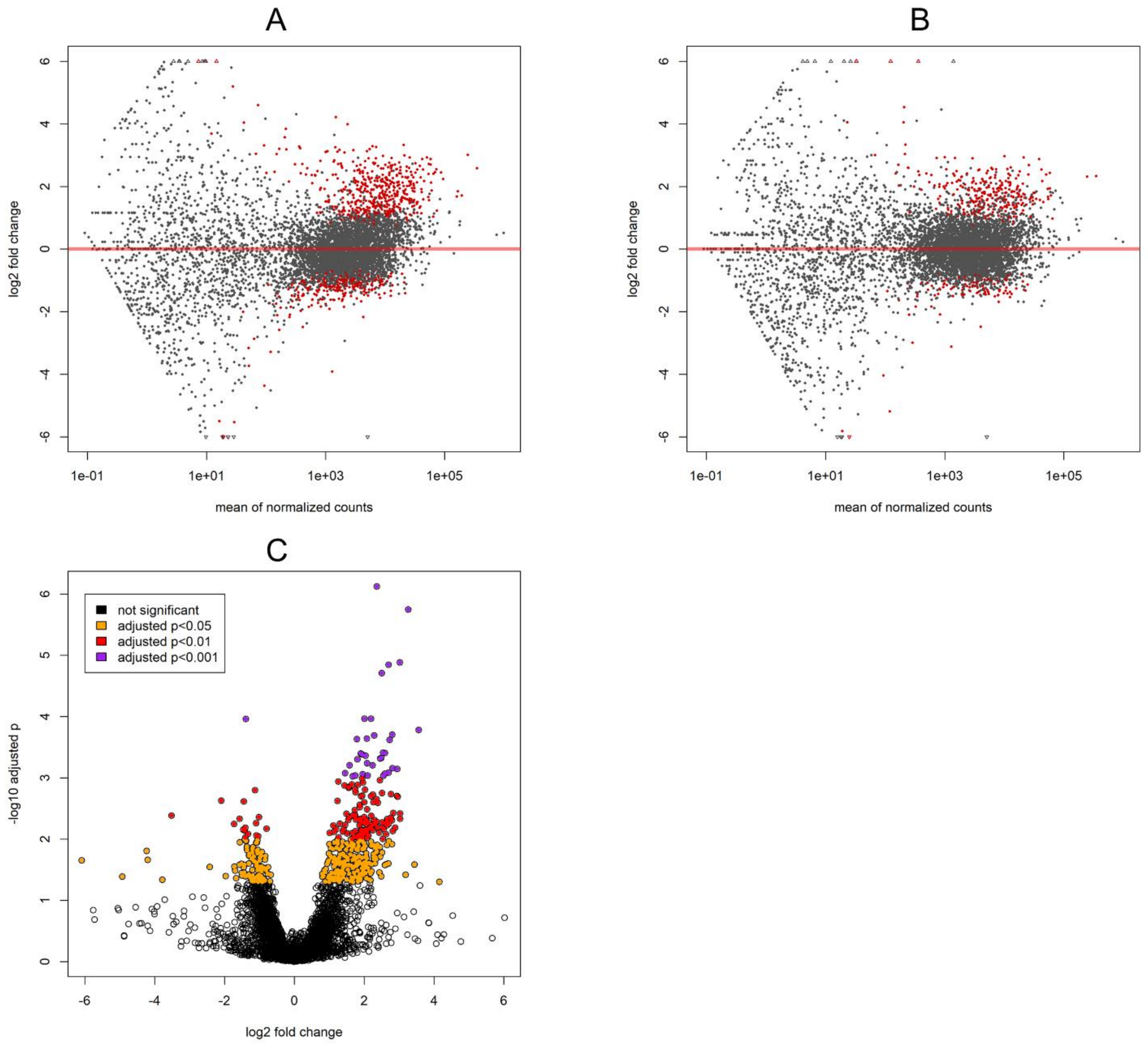

Supplementary Figure 12. (A, B) MA plots representing each gene's log2 fold change (**A**, fold change after comparing the strong and weak inhibition groups; **B**, fold change after comparing the strong and moderate inhibition groups) in relation to the gene's mean expression level (mean of normalized counts). Significant genes with a Benjamini-Hochberg adjusted p-value <0.05 are colored red. **(C)** Volcano plot showing the relationship between fold change and significance level. Fold changes and significance levels (Benjamini-Hochberg adjusted p-values) are the mean of the values obtained for the strong vs weak inhibition and strong vs moderate inhibition group comparisons.

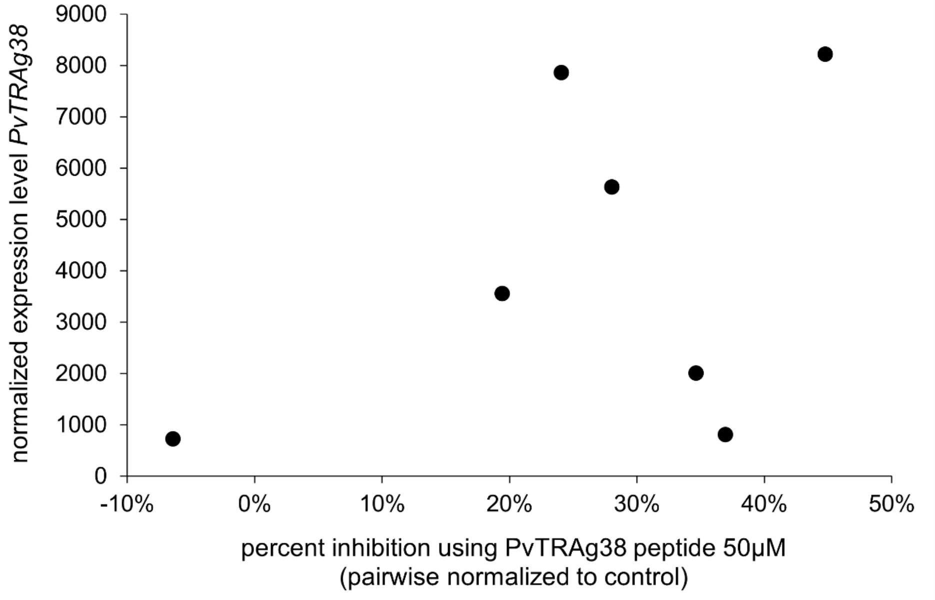

Supplementary Figure 13. Plot showing the normalized expression levels of *PvTRAg38* for the seven isolates used in the invasion assays in the presence of the PvTRAg38 peptide.

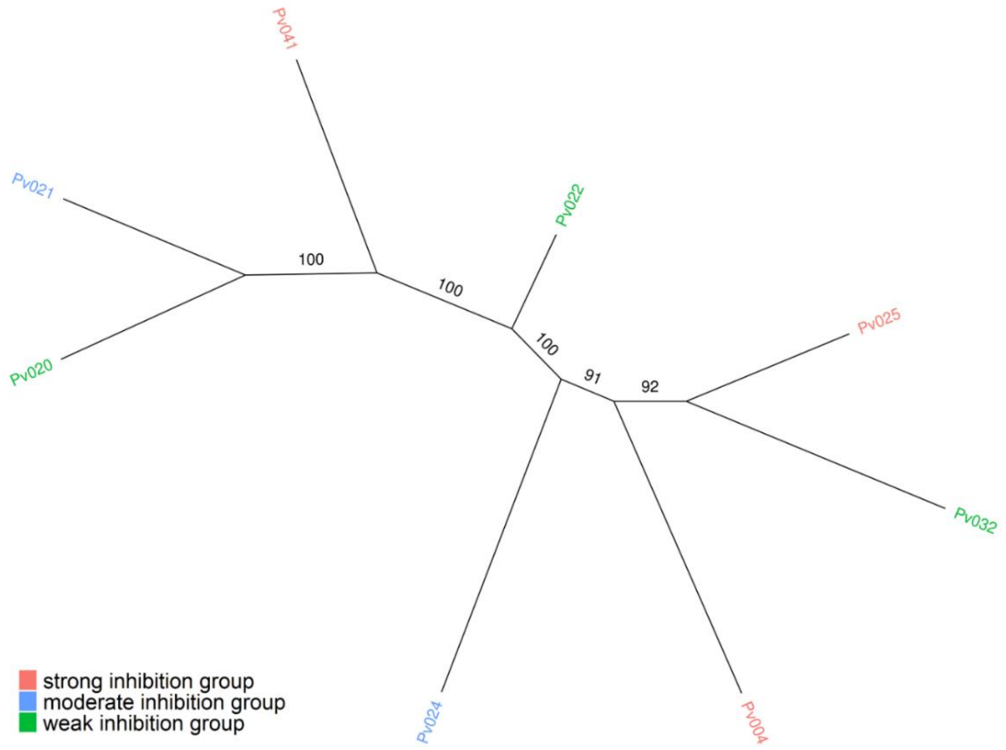

Supplementary Figure 14. Unrooted maximum likelihood tree showing the relatedness between the isolates used for the differential expression analysis, based on WGS data for the core genome (Pearson et al., 2016). Isolates are color-coded based on the differential expression group to which they are assigned. Bootstrap values (%) show the confidence of each branch.

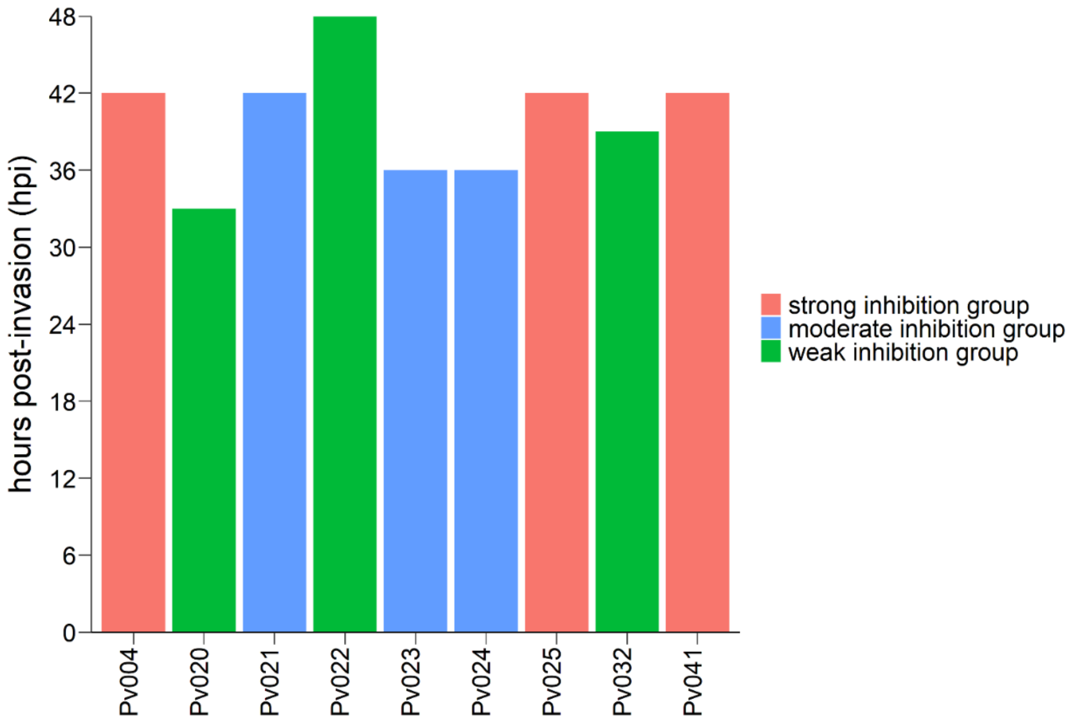

Supplementary Figure 15. Bar plot showing the estimated schizont age (hours post-invasion, hpi) at the moment of harvesting for RNA-Seq for all isolates used in the differential expression analysis. Color codes indicate the differential expression group to which they are assigned. Transcriptomes were Spearman-correlated to the time point–specific transcriptomes from the Zhu et al. (2016) dataset, and the highest correlating time point was considered as the estimated parasite age (hpi) of the RNA-Seq sample.

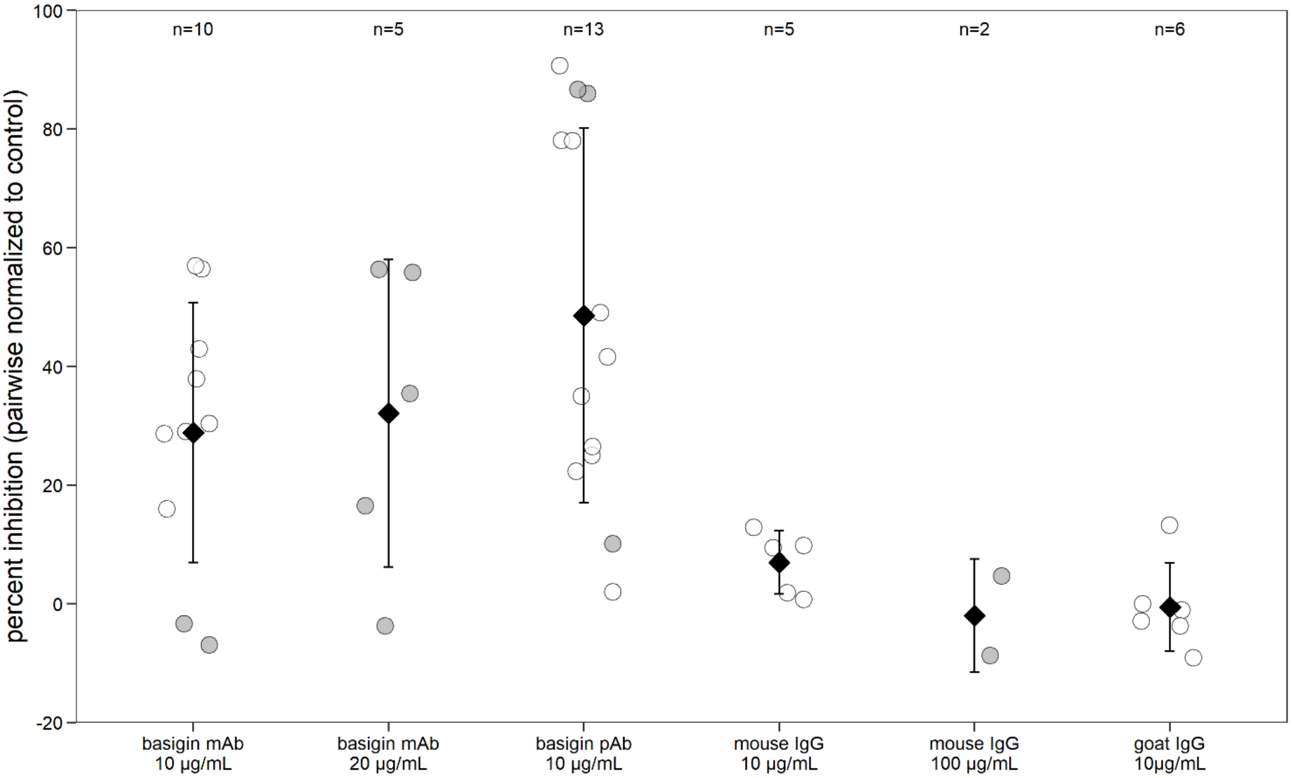

Supplementary Figure 16. *P. vivax* reticulocyte invasion efficiency with basigin blockage, varies across isolates. Dot plot shows *P. vivax* reticulocyte invasion inhibition in invasion assays using anti-basigin monoclonal antibody (mAb; Thermo Fisher Scientific, MA1-10103) and polyclonal antibody (pAb; Bio-Techne, AF972). The invasion inhibition levels are pairwise normalized to the inhibition observed in the control (in absence of mAb or pAb) using the same *P. vivax* isolate. Dots represent individual isolates. Gray dots represent new data, while white dots represent the data shown in Knuepfer et al. (2019) and were processed in the same way. Black diamonds represent the mean percentage of invasion inhibition, with whiskers showing the standard deviation. Mouse IgG 10 µg/mL and 100 µg/mL = basigin mAb positive control (Thermo Fisher Scientific, 31903) , goat IgG 10 µg/mL = basigin pAb positive control (Thermo Fisher Scientific, 31245).
